# Loss of Ryanodine Receptor 2 impairs neuronal activity-dependent remodeling of dendritic spines and triggers compensatory neuronal hyperexcitability

**DOI:** 10.1038/s41418-020-0584-2

**Published:** 2020-07-08

**Authors:** Fabio Bertan, Lena Wischhof, Liudmila Sosulina, Manuel Mittag, Dennis Dalügge, Alessandra Fornarelli, Fabrizio Gardoni, Elena Marcello, Monica Di Luca, Martin Fuhrmann, Stefan Remy, Daniele Bano, Pierluigi Nicotera

**Affiliations:** 1 German Center for Neurodegenerative Diseases (DZNE), Bonn, Germany; 2 Department of Pharmacological and Biomolecular Sciences, Università degli Studi di Milano, Milan, Italy; 3 Department of Cellular Neuroscience, Leibniz Institute for Neurobiology, Magdeburg, Germany

**Keywords:** Neuroscience, Neurological disorders

## Abstract

Dendritic spines are postsynaptic domains that shape structural and functional properties of neurons. Upon neuronal activity, Ca^2+^ transients trigger signaling cascades that determine the plastic remodeling of dendritic spines, which modulate learning and memory. Here, we study in mice the role of the intracellular Ca^2+^ channel Ryanodine Receptor 2 (RyR2) in synaptic plasticity and memory formation. We demonstrate that loss of RyR2 in pyramidal neurons of the hippocampus impairs maintenance and activity-evoked structural plasticity of dendritic spines during memory acquisition. Furthermore, post-developmental deletion of RyR2 causes loss of excitatory synapses, dendritic sparsification, overcompensatory excitability, network hyperactivity and disruption of spatially tuned place cells. Altogether, our data underpin RyR2 as a link between spine remodeling, circuitry dysfunction and memory acquisition, which closely resemble pathological mechanisms observed in neurodegenerative disorders.

## Introduction

Spine number and structural plasticity determine the information storage underlying learning and memory processes [1–5]. Activity-dependent synaptic and structural plasticity of spines require the entry of extracellular Ca^2+^ through N-methyl-D-aspartate receptors (NMDAR) and voltage-gated Ca^2+^ channels (VGCCs) via activation of intracellular signal transduction cascades [6]. The subsequent Ca^2+^ release from the endoplasmic reticulum (ER) initiates a positive feedback mechanism known as Ca^2+^-induced Ca^2+^ release [7]. Ryanodine Receptors (RyRs) are main mediators of Ca^2+^-induced Ca^2+^ release [8–10] with particular relevance for learning and memory processes [11–19]. RyRs play a role in dendritic spine maintenance and plasticity-related remodeling in cultured neurons [11, 19, 20], but the specific in vivo function of RyR subtypes is largely unknown [21].

Among the three RyR isoforms [9, 10], type-2 Ryanodine Receptor (RyR2) is highly expressed in the central nervous system [22] and undergoes activity-dependent transcriptional regulation [23]. RyR2 upregulation has been shown to take place in the hippocampus during spatial memory processes [11, 24]. In addition, an increased content of RyR2 has been demonstrated to occur in response to experimental paradigms of drug-induced plasticity and contextual memory formation [23, 25]. The importance of RyR2 for forebrain-related memory formation has been further supported in various experimental models [18, 26]. Intracranial injection of antisense oligonucleotides against RyR2 leading to global non-cell type-specific suppression of RyR2 expression has been demonstrated to induce transient memory deficits in rodents [27]. In human neurodegenerative disorders with cognitive dysfunction, particularly in Alzheimer’s disease, RyR2 has been shown to be downregulated [28, 29].

Here, we investigated in vitro, ex vivo and in vivo the specific function of RyR2 in the maintenance and activity-dependent structural plasticity of dendritic spines in the post-developmental brain. Our models using both transgenic and AAV-mediated deletion enabled us to specifically investigate the role of RyR2 in the CA1 subfield of the hippocampus, which is of central importance for spatial learning and memory processes [30]. This approach allowed us to identify compensatory mechanisms of neuronal and circuit excitability, which may likely contribute to the dysfunctions found in Alzheimer’s disease where both RyR2 downregulation and altered network excitability has been reliably observed.

## Results

### RyR2 is a regulator of spine density in hippocampal neurons

**Fig. 1:**
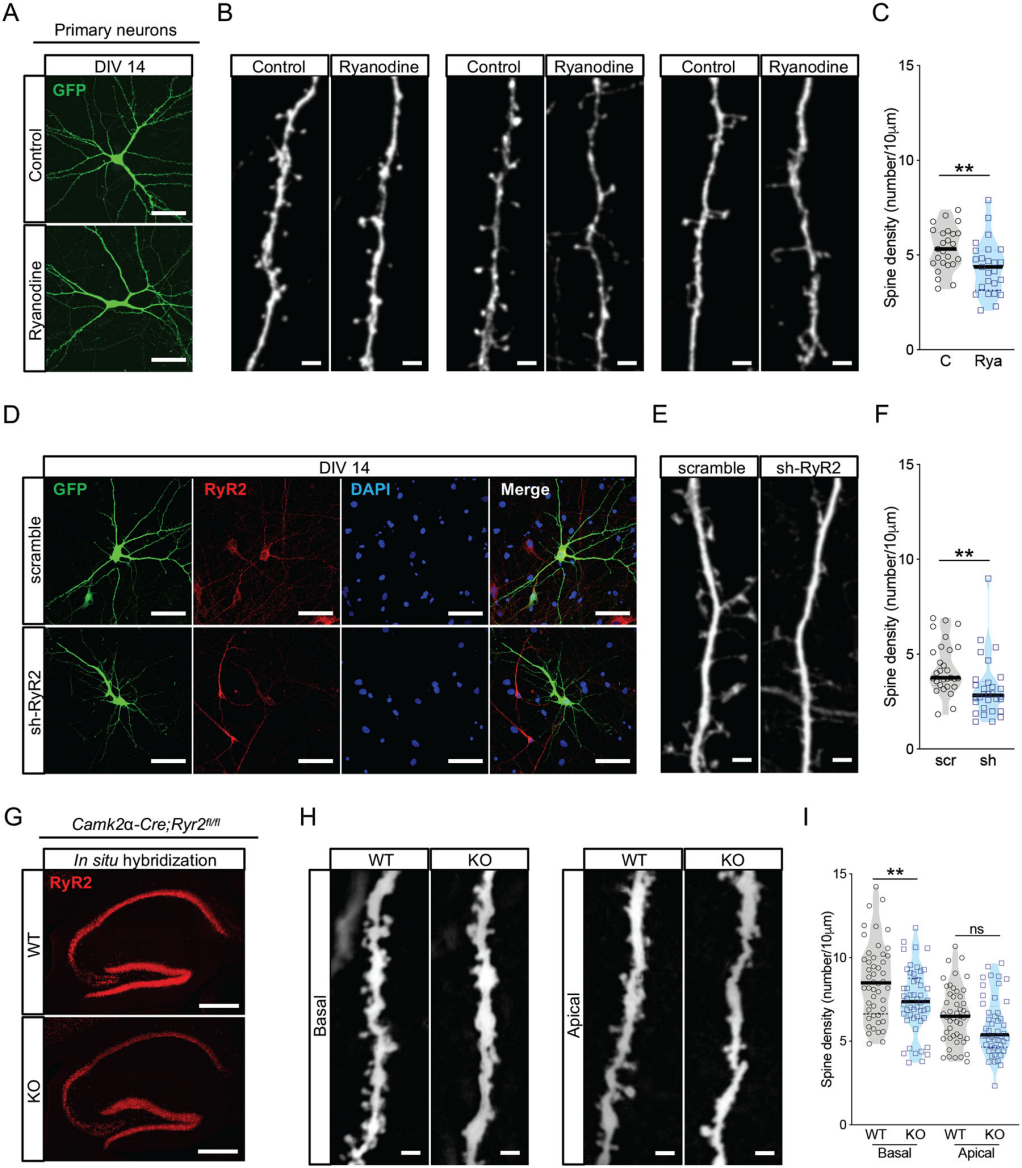
RyR2 mediates maintenance of dendritic spines. **a** Representative images of DIV14 GFP-transfected DMSO (Control) and Ryanodine-treated hippocampal neurons. Scale bar: 50 μm. **b** Representative images of dendritic spines from DMSO (Control) and Ryanodine-treated neurons. Scale bar: 5 μm. **c** Spine density from DMSO (C for Control) and Ryanodine-treated neurons (Rya) (*n* = 3; C: *n* = 25 cells, Rya: *n* = 25 cells). **d** Representative images of immunostained hippocampal neurons transfected with scramble or short-hairpin RNA against *Ryr2* (sh-RyR2). Scale bar: 50 μm. **e** Representative picture of dendritic spines from scramble and sh-RyR2 transfected neurons at DIV14. Scale bar: 5 μm. **f** Quantification of spine density in scramble (scr) and sh-RyR2 (sh) (*n* = 3, scramble: *n* = 29 cells, sh-RyR2: *n* = 30 cells). **g** In situ hybridization for *Ryr2* in sagittal brain sections from control (WT) and *Camk2α-Cre*^*tg/wt*^*;Ryr2*^*fl/fl*^ mice (KO). Scale bar: 400 μm. **h** Representative basal and apical dendrites from CA1 pyramidal neurons in control (WT) and *Camk2α-Cre*^*tg/wt*^*;Ryr2*^*fl/fl*^ mice (KO). Scale bar: 2 μm. **i** Quantification of spine density in basal (WT: *n* = 48 cells/5 mice, KO: *n* = 50 cells/5 mice) and apical (WT: *n* = 47 cells/5 mice, KO: *n* = 48 cells/5 mice) dendrites of CA1 neurons in control (WT) and *Camk2α-Cre*^*tg/wt*^*;Ryr2*^*fl/fl*^ mice (KO). Data are reported as median [25th and 75th percentile]. Unpaired Student’s *t* test, **p < 0.01.

First, we tested the effect of the irreversible RyRs antagonist Ryanodine on spine density regulation in primary neuronal cultures. Hippocampal neurons were transfected with a vector encoding GFP at day in vitro 8 (DIV8) and treated for 60 minutes with DMSO (control) or Ryanodine (50 μM) at DIV14 (Fig. 1a). Ryanodine treatment decreased dendritic spine number (18.7% reduction, Fig. 1b, c; for statistics see Table S1), confirming the involvement of RyRs in the maintenance of dendritic spines [11, 20]. Next, we downregulated RyR2 in hippocampal primary cultures (Fig. 1d). Compared to control (scramble), short hairpin RNA against *Ryr2* (sh-RyR2) led to a markedly decreased spine density (25.6% reduction, Fig. 1e, f; for statistics see Table S1). These data suggested that RyR2 may play a role in the regulation of spine density in vivo. Thus, to gain a first general insight into the role of RyR2 during development and in activity-dependent in vivo processes, we generated RyR2 conditional knockout mice (Supplementary Fig. S1A–C). We bred *Ryr2*^*fl/fl*^ mice with either *Synapsin-Cre*^*tg/wt*^ (Supplementary Fig. S2A–L) for neuron-specific knockout or *Camk2α-Cre*^*tg/wt*^ (Fig. 1g) for forebrain-restricted knockout with predominant action on pyramidal cell populations. We found that *Synapsin-Cre*^*tg/wt*^*;Ryr2*^*fl/fl*^ mice exhibited reduced spine density in the hippocampus compared to control littermates (9.75% reduction, Supplementary Fig. S2J–L; for statistics see Table S1). In addition, we observed that neuronal-specific knock-out in *Synapsin-Cre*^*tg/wt*^*;Ryr2*^*fl/fl*^ mice resulted in locomotor defects (Supplementary Fig. S2A–I; for statistics see Table S2). When we restricted the *Ryr2* deletion to the forebrain (*Camk2α-Cre*^*tg/wt*^*;Ryr2*^*fl/fl*^), the postnatal (i.e., around P17.5) loss of RyR2 in excitatory neurons did not affect either locomotor activity or motor coordination (Supplementary Fig. S3A–C; for statistics see Table S2). Again, we reliably observed a reduced spine number in CA1 neurons when we compared control mice with *Camk2α-Cre*^*tg/wt*^*;Ryr2*^*fl/fl*^ mice (Fig. 1h, i; for statistics see Table S1). This spine density reduction was mostly pronounced at the basal dendritic tree of hippocampal CA1 pyramidal neurons (14% reduction, Fig. 1h, i; for statistics see Table S1). These data obtained in different model systems consistently demonstrate a role of RyR2 function in spine homeostasis of hippocampal neurons.

### RyR2 function is required for activity-dependent structural plasticity of spines during memory processes

**Fig. 2:**
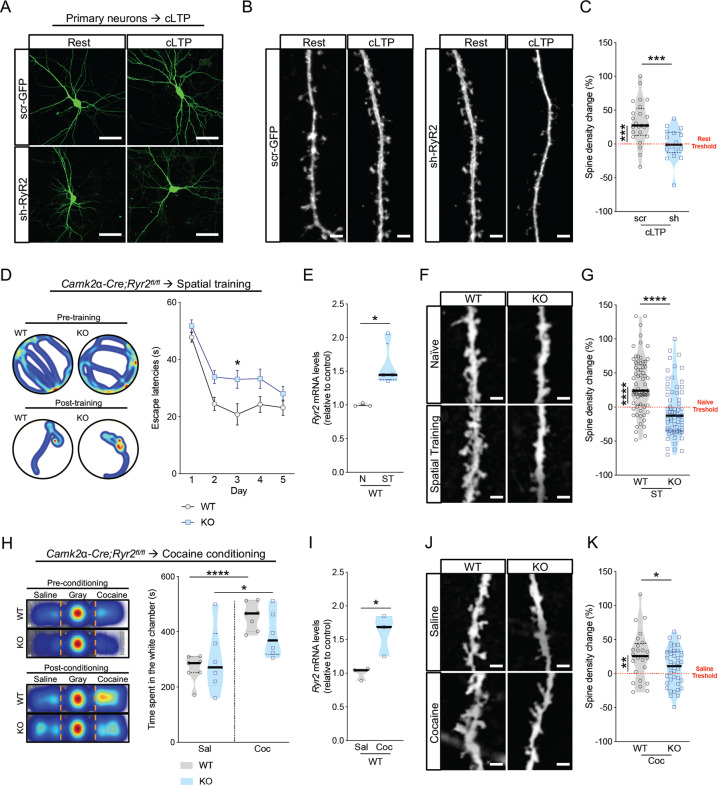
RyR2 mediates activity-dependent structural plasticity of dendritic spines in the hippocampus. **a**, **b** Representative pictures of transfected scramble and sh-RyR2 DIV14 neurons kept in resting condition or treated with cLTP. Neurons, scale bar: 50 μm. Dendritic spines, scale bar: 2 μm. **c** Spine density change in scramble (scr) and sh-RyR2 (sh) neurons treated with cLTP and compared to cells at rest (*n* = 3, scramble: *n* = 29 cells, scramble cLTP: *n* = 24 cells, sh-RyR2: *n* = 30 cells, sh-RyR2-cLTP: *n* = 19 cells). **d**–**g** Control (WT) and *Camk2α-Cre*^*tg/wt*^*;Ryr2*^*fl/fl*^ mice (KO) trained in the Morris Water Maze, a paradigm of hippocampal structural plasticity. **d** Heatmaps and escape latencies over training days in the Morris Water Maze of control (WT) and *Camk2α-Cre*^*tg/wt*^*;Ryr2*^*fl/fl*^ (KO) mice (WT: *n* = 13, KO: *n* = 18). **e** qRT-PCR of *Ryr2* mRNA expression of hippocampal samples from naïve- (N) and spatial trained- (ST) control mice (WT naïve: *n* = 3, WT spatial trained: *n* = 4). **f**, **g** Spine density change in apical dendrites (WT naïve: *n* = 47 cells/5 mice, WT spatial trained: *n* = 69 cells/7 mice, KO naïve: *n* = 48 cells/5 mice, KO spatial trained: *n* = 66 cells/7 mice) of CA1 neurons induced by spatial training (24 h after the last training) in control (WT) and *Camk2α-Cre*^*tg/wt*^*;Ryr2*^*fl/fl*^ (KO) mice compared to naïve control (WT) and *Camk2α-Cre*^*tg/wt*^*;Ryr2*^*fl/fl*^ (KO) mice. Scale bar: 2 μm. **h**–**k** Control (WT) and *Camk2α-Cre*^*tg/wt*^*;Ryr2*^*fl/fl*^ (KO) mice subjected to cocaine-induced conditioned place preference (CPP) test, a second paradigm of hippocampal structural plasticity. (H) Heatmaps and contextual place preference score of the time spent in the CPP apparatus (white chamber) pre- and post- 6 days of alternated cocaine conditioning using control (WT) and *Camk2α-Cre*^*tg/wt*^*;Ryr2*^*fl/fl*^ (KO) mice (WT saline: *n* = 7, WT cocaine: *n* = 7, KO saline: *n* = 6, KO cocaine: *n* = 6). **i** qRT-PCR of *Ryr2* mRNA expression of hippocampal samples from saline- (Sal) and cocaine- (Coc) treated control mice (WT saline: *n* = 3, WT cocaine: *n* = 3). **j**, **k** Spine density change 24 h after the post-conditioning test in apical dendrites (WT naïve: *n* = 23 cells/3 mice, WT cocaine: *n* = 24 cells/4 mice, KO naïve: *n* = 32 cells/4 mice, KO cocaine: *n* = 47 cells/6 mice) of CA1 neurons in cocaine (Coc)-treated control (WT) and *Camk2α-Cre*^*tg/wt*^*;Ryr2*^*fl/fl*^ (KO) mice compared to saline-treated control (WT) and *Camk2α-Cre*^*tg/wt*^*;Ryr2*^*fl/fl*^ (KO) mice. Scale bar: 2 μm. Data are reported as median [25th and 75th percentile]. Unpaired Student’s *t* test or Two-way RM ANOVA with Bonferroni post hoc comparison, ****p < 0.0001, ***p < 0.001, *p < 0.05.

Next, we hypothesized that RyR2 function may be required for activity- and experience-dependent spine formation during memory processes. We initially used hippocampal cultures transfected with either scramble or sh-RyR2. Neuronal plasticity was triggered using a cocktail of compounds (i.e., forskolin, picrotoxin and rolipram), which is known to induce chemical LTP (cLTP; Fig. 2a–c; for statistics see Table S1) [31, 32]. Chemical LTP increased spine density (32.1% increase) in scramble cells, while no increase was observed in RyR2 knockdown neurons (Fig. 2a–c; for statistics see Table S1). Thus, we found that RyR2 mediates activity-dependent structural plasticity of dendritic spines following cLTP induction.

We next performed two hippocampal-dependent plasticity-inducing behavioral paradigms in *Camk2α-Cre*^*tg/wt*^*;Ryr2*^*fl/fl*^ mice: (a) spatial memory acquisition in the Morris Water Maze (MWM) [33] and (b) a cocaine-induced conditioned place preference paradigm (CPP) that induces spatial and contextual memory formation [34]. In the MWM paradigm mice were trained for five days (see Material and Methods) during which we monitored escape latencies as a measure of training performance (Fig. 2d). While *Camk2α-Cre*^*tg/wt*^*;Ryr2*^*fl/fl*^ animals initially showed a slower performance improvement and a mildly increased escape latency (Fig. 2d; for statistics see Table S2), they performed similar to control littermates by the end of the training period. This result was consistent throughout different short- and long- term memory tests (Supplementary Fig. S3D–H; for statistics see Table S2). Then, we investigated the effects of spatial training on RyR2 levels and spine formation in hippocampal neurons. In agreement with previous studies [11, 24], spatial memory acquisition produced an upregulation of *Ryr2* in the hippocampus (Fig. 2e; unpaired t-test, *p* = 0.0303). In parallel, MWM training induced an increased spine number in control mice as shown by *postmortem* golgi staining of excitatory CA1 neurons. Interestingly, this effect was mostly restricted to apical dendrites (32.6% increase, Fig. 2f, g; for statistics see Table S1), but not at basal projections of pyramidal cells (6.5% increase, Supplementary Fig. S4A, B; for statistics see Table S1). Strikingly, RyR2 deficient *Camk2α-Cre*^*tg/wt*^*;Ryr2*^*fl/fl*^ mice did not exhibit changes in spine number neither on apical (by −5.6%, Fig. 2f, g; for statistics see Table S1) nor basal dendrites (11.4% reduce, Supplementary Fig. S4A, B; for statistics see Table S1), when compared to naïve *Camk2α-Cre*^*tg/wt*^*;Ryr2*^*fl/fl*^ mice. In line with our hypothesis, these data demonstrate that spatial training-evoked spine remodeling of CA1 neurons requires RyR2.

As a second paradigm of plasticity, we performed cocaine-induced conditioned place preference paradigm (CPP). We selected this paradigm, because we have shown previously that repeated treatment with cocaine results in the upregulation of *Ryr2* in the hippocampus of rodents [23], an event associated with the plastic remodeling of synaptic circuits [35]. In the CPP test, mice were trained to associate the stimulating effect of cocaine with a paired context (e.g., white chamber) in comparison to a distinct saline-paired context (e.g., dark chamber) (Fig. 2h). *Camk2α-Cre*^*tg/wt*^*;Ryr2*^*fl/fl*^ and control mice were first habituated to the three-chamber CPP box where they did not show any initial preference for either context (i.e., the black or white chamber of the CPP box). Thereafter, *Camk2α-Cre*^*tg/wt*^*;Ryr2*^*fl/fl*^ and control mice were conditioned over 6 days through alternate injections of either saline or cocaine (20 mg/kg). Following the conditioning phase, the strength of the cocaine-associated memory was then tested by measuring the time that the animals spent in the cocaine- over the saline-paired compartment. Both, control and *Camk2α-Cre*^*tg/wt*^*;Ryr2*^*fl/fl*^ mice exhibited a preference for the cocaine-paired compartment (Fig. 2h; for statistics see Table S2), although *Camk2α-Cre*^*tg/wt*^*Ryr2*^*fl/fl*^ mice showed a significantly lower time spent in the cocaine-paired chamber (Supplementary Fig. S3I; for statistics see Table S2), suggesting a function of RyR2 in cocaine-associated memory formation. Treatment with cocaine produced an upregulation of *Ryr2* in the hippocampus (Fig. 2i; unpaired t-test, *p* = 0.0334) in agreement with our previously published data [23]. After 24 h from the CPP test, saline- and cocaine-treated RyR2 knockout and control littermates were sacrificed for a *postmortem* assessment of the dendritic spine density in CA1 pyramidal neurons using golgi staining. As shown for the spatial training, cocaine-treated control mice exhibited a significant increase in spine number when compared to saline-treated control mice. This spine number change occurred predominantly in apical dendrites (25.9% increase, Fig. 2j, k; for statistics see Table S1) and with less magnitude in basal dendrites (15.6% increase, Supplementary Fig. S4C, D; for statistics see Table S1). Cocaine-treated *Camk2α-Cre*^*tg/wt*^*;Ryr2*^*fl/fl*^ mice exhibited only a mild increase of spine number in apical dendrites compared to saline-treated *Camk2α-Cre*^*tg/wt*^*;Ryr2*^*fl/fl*^ mice (9.1% increase, Fig. 2j, k; for statistics see Table S1). In basal dendrites and compared to saline-treated *Camk2α-Cre*^*tg/wt*^*;Ryr2*^*fl/fl*^ mice, cocaine-treated *Camk2α-Cre*^*tg/wt*^*;Ryr2*^*fl/fl*^ mice exhibited an increased spine number change, which was comparable to control cocaine-treated mice (14.2% increase, Supplementary Fig. S4C, D; for statistics see Table S1). Overall, our data demonstrate that RyR2 knockout impairs the activity-depended formation of dendritic spines in different plasticity-induced behavioral paradigms.

### Acute RyR2 knockout leads to reduced spine densities, reduced excitatory synaptic input and compensatory cellular hyperexcitability

**Fig. 3:**
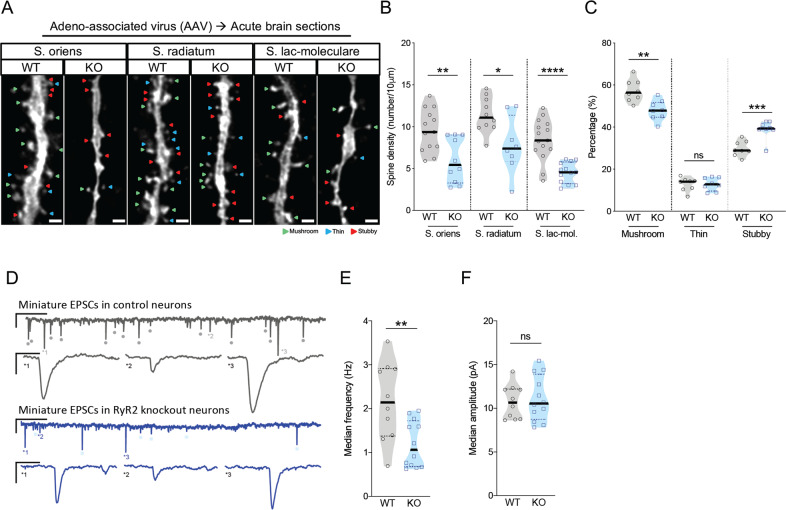
Post-developmental RyR2 deletion leads to loss of excitatory synapses. **a** Representative images of dendrites of stratum oriens, stratum radiatium and stratum lacunosum-moleculare of stereotaxically injected *AAV.Camk2α.GFP* (control, WT) and *rAAV.Camk2α.GFP-cre* (RyR2 knockout, KO) mice. Different spine morphologies: mushroom (green), thin (blue) and stubby (red). Scale bar: 1 μm. **b** Spine density in dendrites of the stratum s. oriens (WT: *n* = 13 cells/9 mice, KO: 10 cells/5 mice), s. radiatum (WT: *n* = 10 cells/7 mice, KO: 8 cells/4 mice) and s. lacunosum-moleculare (WT: *n* = 15 cells/9 mice; KO: 13 cells/6 mice). **c** Percentage of mushroom, thin and stubby dendritic spines in control (WT) and RyR2 knockout (KO) pyramidal neurons (WT: *n* = 8 cells/6 mice, KO: 8 cells/6 mice). **d** Representative examples of miniature EPSCs measurements in control and RyR2 knockout CA1 pyramidal cells. Scale bars: 10pA, 1 s and 10 pA, 25 ms. **e**, **f** Summary of the measured miniature EPSCs in control (WT) and RyR2 knockout (KO) neurons (WT: *n* = 10 cells/4 mice/7821 EPSCs, KO: 12 cells/5 mice/5057 EPSCs) for **e** the instantaneous EPSC frequency and **f** the EPSC amplitude. Data are reported as median [25th and 75th percentile]. Unpaired Student’s *t* test or Two-way ANOVA with Bonferroni post hoc comparison. ****p < 0.0001, ***p < 0.001, **p < 0.01, *p < 0.05.

RyR2 has been shown to mediate dendritic spine maturation and clustering during development [36]. Since the genetic ablation of *Ryr2* in *Camk2α-Cre*^*tg/wt*^ mice starts from the third post-natal week [37], the role of RyR2 in adulthood might have been obscured by compensatory developmental mechanisms. Therefore, we applied a viral-vector based strategy for RyR2 knockout in adult mice, which in addition restricted the deletion to hippocampal subfield CA1. This approach enabled us to investigate the acute effects of RyR2 deletion on spine density gaining insight into putative compensatory mechanisms. Since altered regulation of RyR2 expression has been observed in pathological conditions such as Alzheimer’s disease [28, 38, 39], the observation of compensatory mechanisms on the level of neuronal excitability, neuronal morphology and network function might provide important pathophysiological insights. Thus, we unilaterally injected either *rAAV.CamK2α.GFP-cre* or *AVV.CamK2α.GFP* (as control) in the CA1 region of the hippocampus of 5-month-old *Ryr2*^*fl/fl*^ mice (Supplementary Fig. S5A). In-situ hybridization confirmed the genetic deletion of *Ryr2* mRNA in GFP-positive cells 4–6 weeks after the AAV injection (Supplementary Fig. S5B). We performed a detailed morphometric analysis on the level of dendritic spines in biocytin-reconstructed CA1 pyramidal cells (Fig. 3a–c). In addition, this approach in acute hippocampal slices allowed for an electrophysiological assessment of neuronal connectivity and excitability. In full agreement with our previous results in *Camk2α-Cre*^*tg/wt*^*;Ryr2*^*fl/fl*^ mice we found that spine density of CA1 pyramidal cells was significantly decreased in RyR2 knockout neurons (39.1% reduce, Table 1). Spine density reduction was observed within the s. oriens, s. radiatum and s. lacunosum-moleculare (Fig. 3a, b and Table 1). Furthermore, spine morphology was classified as mushroom, stubby or thin [40]. The number of mature mushroom spines was overall reduced by about 9.4% while the number of less mature stubby spines was increased by 9.1% in RyR2 knockout neurons when compared to control cells (Fig. 3c and Table 1). Thin spines were unchanged (Fig. 3a, b and Table 1). Taken together, we found that loss of mushroom spines occurred within dendrites of s. oriens and s. lacunosum-moleculare, whereas no differences in spine shape distribution were observed in radial oblique dendrites (Table 1). Thus, the acute depletion of RyR2 resulted in a predominant loss of matured spines in distal dendritic compartments. Having electrophysiological access to individual cells allowed us to determine excitatory synaptic connectivity by recording TTX-insensitive miniature excitatory postsynaptic currents (mEPSCs) (Fig. 3d–f). The frequency of mEPSCs was reduced in RyR2 knockout CA1 pyramidal neurons (Fig. 3d; WT: 2.12 ± 0.89 Hz vs. KO: 1.19 ± 0.53 Hz; unpaired t test, *p* = 0.0048), while the mEPSCs amplitude (Fig. 3f; WT: 10.72 ± 1.94 pA vs. KO: 11.1 ± 2.59 pA; *p* = 0.7041, unpaired t test) was unchanged when compared to control cells. This finding is consistent with a reduced number of excitatory synapses in RyR2 knockout neurons and in full agreement with the observed reduction of dendritic spines.

**Table 1:** Statistical analysis of hippocampal CA1 neurons reconstructed from *RyR2*^*fl/fl*^ mice transfected with *rAAV.CamK2α.GFP* (WT) vs *rAAV.CamK2α.cre-GFP* (KO).

Neurons	WT	KO	*p* value	Sum
**Basal dendrites**				
Length (μm)	2268 ± 185.5	1381 ± 149.2	0.0006	***
Surface area (μm^2^)	14672 ± 1223	8700 ± 905.1	0.0003	***
Volume (μm^3^)	8391 ± 743.5	4859 ± 554.1	0.0004	***
Dendritic thickness (μm)	2.193 ± 0.04045	2.04 ± 0.04509	0.0173	*
Number branch points	35.44 ± 3.58	21.14 ± 2.925	0.0035	**
**Apical dendrites**				
Length (μm)	2938 ± 271.3	2386 ± 161.5	0.0729	ns
Surface area (μm^2^)	20193 ± 1724	15494 ± 1205	0.0277	*
Volume (μm^3^)	12042 ± 992.9	8749 ± 779.9	0.0128	*
Dendritic thickness (μm)	2.318 ± 0.05193	2.159 ± 0.0321	0.0100	*
Number branch points	34.5 ± 4.863	32.1 ± 3.8	0.6964	ns
**Total**				
Length (μm)	4810 ± 461.6	3335 ± 282.5	0.0100	*
Surface area (μm^2^)	32122 ± 2921	22280 ± 2032	0.0097	**
Volume (μm^3^)	18767 ± 1674	12936 ± 1403	0.0133	*
Dendritic thickness (μm)	2.264 ± 0.05678	2.08 ± 0.04186	0.0197	*
Number branch points	65.55 ± 9.009	44.54 ± 5.7	0.0543	ns

Spines	WT	KO	*P* value	Sum
**S. oriens**				
Density	9.556 ± 0.7015	6.004 ± 0.842	0.0037	**
% Mushroom	59.47 ± 1.652	46.64 ± 2.726	0.0005	***
% Thin	10.18 ± 0.9954	8.701 ± 1.229	0.3569	ns
% Stubby	30.25 ± 1.579	44.45 ± 3.074	0.0003	***
Spine length (μm)	1.217 ± 0.0193	1.095 ± 0.0254	0.0001	***
**S. radiatum**				
Density	11.4 ± 0.6692	7.797 ± 1.195	0.0137	*
% Mushroom	56.86 ± 2.124	53.98 ± 2.891	0.4356	ns
% Thin	12.01 ± 1.481	12.8 ± 1.381	0.7026	ns
% Stubby	31.13 ± 1.428	33.22 ± 2.051	0.4170	ns
Spine length (μm)	1.224 ± 0.02199	1.219 ± 0.0227	0.8663	ns
**S. lacunosum moleculare**				
Density	8.444 ± 0.6342	4.529 ± 0.34	<0.0001	****
% Mushroom	55.99 ± 2.851	46.88 ± 3.025	0.0386	*
% Thin	18.14 ± 1.475	15.45 ± 1.878	0.2639	ns
% Stubby	25.87 ± 2.218	37.32 ± 3.003	0.0046	**
Spine length (μm)	1.396 ± 0.02258	1.29 ± 0.03129	0.0068	**
**Total**				
Density	9.602 ± 0.425	5.848 ± 0.4809	<0.0001	****
% Mushroom	57.32 ± 1.865	47.96 ± 1.674	0.0022	**
% Thin	12.84 ± 1.126	12.88 ± 1.1	0.9771	ns
% Stubby	29.79 ± 1.268	38.9 ± 1.568	0.0005	***

**Fig. 4:**
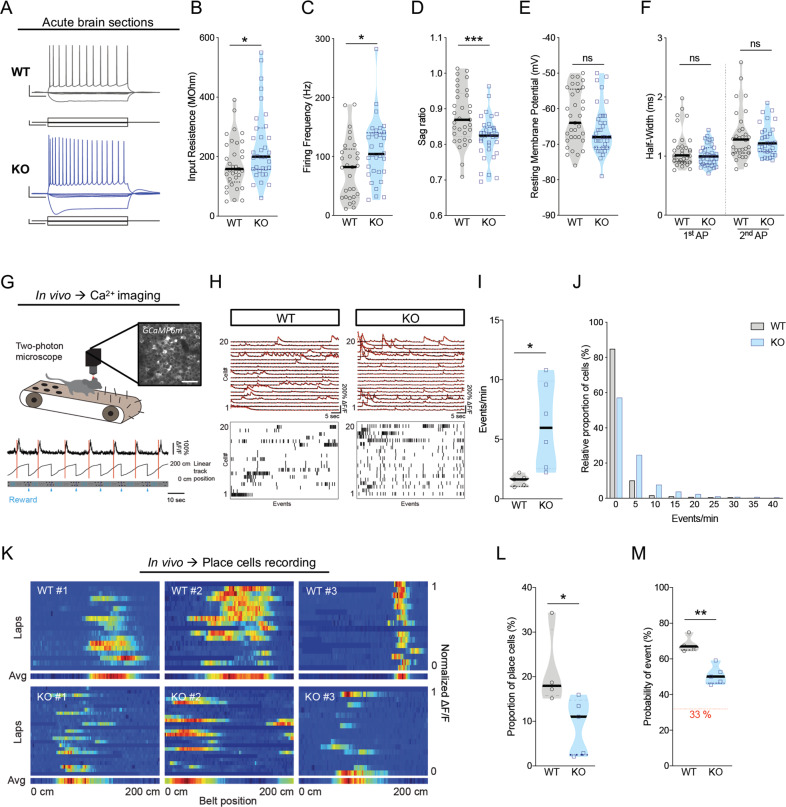
Acute deletion of RyR2 in the adult brain leads to compensatory cellular hyperexcitability and dendritic sparsification. **a**–**f** Intrinsic cell firing properties in acute brain sections obtained from *Ryr2*^*fl/fl*^ mice stereotaxically injected with *AAV.Camk2α.GFP* (control, WT) or with *rAAV.Camk2α.GFP-cre* (RyR2 knockout, KO) in the hippocampus. **a** Representative examples of firing properties in control (WT) and RyR2 knockout (KO) CA1 pyramidal neurons. Electrophysiological assessment of (**b**) input resistance, (**c**) firing frequency, (**d**) sag ratio, (**e**) resting membrane potential and (**f**) half-width in control (WT) and *Ryr2* knockout (KO) neurons (WT: *n* = 33 cells/11 mice, KO: *n* = 34 cells/10 mice). **g** In vivo Ca^2+^ imaging of control C57BL/J6 and *Ryr2*^*fl/fl*^ mice stereotactically injected with both *rAAV9.CamK2α-cre* and *rAAV1.Syn.Flex.GCaMP6m*. **h** Recording of Ca^2+^ signals in CA1 neurons of control (WT) and RyR2 knockout (KO) mice at rest. Upper panel: Overlay of 20 exemplary ΔF/F-traces (black) and denoised traces (red) events. Lower panel: deconvoluted spikes. **i** Average frequency of Ca^2+^ events in CA1 neurons in control (WT) and RyR2 knockout (KO) mice (WT: *n* = 5, KO: *n* = 6). **j** Frequency distribution of all recorded cells (WT: *n* = 723. KO: *n* = 2706). **k** Three exemplary heatmaps of identified spatially tuned cells for control (WT) and RyR2 knockout (KO) mice trained on a linear treadmill with tactile cues. **l** Comparison of proportion of identified place cells in control (WT) and RyR2 knockout (KO) mice (WT: *n* = 4, KO: *n* = 5). **m** Comparison of the probability of a significant Ca^2+^ event when mouse passes through place field for control (WT) and RyR2 knockout (KO) mice (WT: *n* = 4, KO: *n* = 5). Data are reported as median [25th and 75th percentile]. Student *t* test or Mann–Whitney test. ***p < 0.001, **p < 0.01, *p < 0.05.

We then assessed the electrical properties of CA1 pyramidal cells by somatic current injections in whole-cell current-clamp mode. We found that neurons responded to RyR2 deletion by an increase of intrinsic excitability (Fig. 4a–f). The input resistance was increased by approximately 34% (Fig. 4b; WT: 158.6 [115.1; 243.1] MΩ, KO: 199.9 25 [156.6; 296.4] MΩ; Mann–Whitney test, *p* = 0.0357). As a result, neuronal firing rates in response to current injection were increased (Fig. 4c; WT: 82.03, [31.16; 112.8] Hz, KO: 104.2 [73.95; 140.2] Hz; Mann–Whitney test, *p* = 0.0243). Moreover, the sag ratio was significantly decreased (Fig. 4d; WT: 0.8693 [0.8215; 0.9438], KO: 0.8247 [0.7855; 0.8484]; Mann–Whitney test, *p* = 0.0008) indicating a stronger activation of hyperpolarization-activated cyclic nucleotide-gated ion channels (HCN). We did not observe a difference, neither in the resting membrane potential measured immediately after establishing the whole-cell mode, nor in the waveform of the first or second action potential duration (Fig. 4e, f). All changes were predictive of a reduced neuronal size due to reduced dendritic length, which would be consistent with higher neuronal input resistance, higher firing rate and more effective recruitment of hyperpolarization-activated dendritic channels [41, 42]. Sparsification of dendritic branches is a hallmark of several neurodegenerative diseases and can be reliably observed in postmortem tissue of Alzheimer patients as well as rodent disease models [42]. To test this, we performed a morphometric analysis of the biocytin-reconstructed neurons (Supplementary Fig. S4A and Table 1). Compared to controls, this analysis revealed a significant decrease of the dendritic length, diameter, branching, area and volume measured in all the CA1 strata of RyR2 knockout cells (Table 1). Moreover, basal dendrites of the stratum oriens were significantly reduced in length, diameter, area, volume and branch points (Table 1). The apical dendritic tree showed less pronounced differences predominantly in area, volume and diameter when compared to CA1 pyramidal cells from mice injected with *rAVV.CamK2α.GFP* (Table 1). Comprehensive statistical analysis revealed that RyR2 knockout neurons were ~30.7% shorter in length, ~30.6% smaller in area, ~31.1% smaller in volume and ~8.1% shorter in diameter (Table 1). These morphological alterations indicate a neuronal shrinkage, which is associated with an enhanced cellular excitability of RyR2 knockout CA1 pyramidal neurons.

Finally, we investigated in vivo the physiological consequences of RyR2 knockout at the level of neuronal spatial tuning and network function. We used two-photon Ca^2+^-imaging in the hippocampus of awake mice to monitor Ca^2+^ transients in CA1 pyramidal neurons (Fig. 4g–m). Five months old *Ryr2*^*fl/fl*^ and age-matched control mice were bi-laterally injected with *rAAV9.CamK2α-cre* and *rAAV1.Syn.Flex.GCaMP6m* into the dorsal hippocampus. A chronic hippocampal window implant [43, 44] allowed us to gain access to the CA1 cell layer. Ca^2+^-imaging of CA1 pyramidal neurons was carried out in head-fixed awake mice running on a linear treadmill with different tactile textures (Fig. 4g and Materials and Methods section) [45]. To test whether RyR2 deletion had an effect on neuronal activity patterns and tuning, we analyzed the Ca^2+^ transient frequency during rest in awake mice (Fig. 4h–j) using the OASIS toolbox [46]. In agreement with the increased neuronal excitability observed in the slice recordings, we found a significantly higher Ca^2+^ transient frequency in RyR2 knockout compared to control mice (Fig. 4i; WT: 1.6 [1.1;2] events/min, KO: 6.2 [2.5;9.9] events/min, Mann–Whitney test, *p* = 0.0043). We also observed a shift of the event distribution towards higher event frequencies (Fig. 4j). We next investigated, whether RyR2 knockout affected the spatial tuning of CA1 neurons (Fig. 4k). We next recorded place cell activity in the two experimental groups (for inclusion criteria of place cells see Materials and Methods). Within a total population of 575 neurons, we identified a total of 121 place cells in 4 control animals compared to 132 place cells in a total of 1776 cells in 5 RyR2 knockout mice. We compared the fraction of place cells of all active cells and found a significant decrease of place cells in RyR2 knockout mice (Fig. 4l; WT: 11 [3; 15]%, KO: 18 [16; 30]%, Mann–Whitney test, *p* = 0.0317). Furthermore, the probability of an observed place cell response during place field passage was significantly decreased in RyR2 knockout compared to control mice (Fig. 4m; WT: 67 [65; 73]%, KO: 50 [46; 56]%, Mann–Whitney test, *p* = 0.0159). To test, whether the place cell deficits correlated with spatial memory deficits, animals were tested in an eight-arm maze (Supplementary Fig. S6A–H; for statistics see Table S2). RyR2 knockout mice displayed a significant impairment in learning to find the baits compared to control mice, further supporting the role of RyR2 in hippocampus-dependent memory processes (Supplementary Fig. S6F–H; for statistics see Table S2). In summary, these data support the role of the RyR2 in regulating spine density and excitatory input. Interestingly, the compensatory changes we found on the level of cellular excitability, neuronal morphology, spatial neuronal tuning and memory resemble the pattern of pathological changes seen in several neurodegenerative diseases [42, 47–50] in which changes of RyR2 have been described.

## Discussion

Taken together, we demonstrated that RyR2 regulates spine number, synaptic connectivity and activity-dependent structural plasticity associated with hippocampal memory processes in adult brain. Importantly, loss of RyR2 led to compensatory changes resulting in increased cellular excitability, altered neuronal morphology and impaired neuronal tuning that closely resembled pathomechanisms of circuit dysfunction in neurodegenerative disorders [42, 47–50].

Until now, the consequences of RyR2 loss have been investigated either ex vivo or in vivo using pharmacology and transient injection of downregulating oligonucleotides (OGNs) [11–13, 15, 18–20, 23, 36, 51, 52]. An in vivo investigation of RyR2 function in neurons of the post-developmental brain was still absent. Building up on the in vitro findings that RyR2 is important for dendritic spine homeostasis [11, 19] and the in vivo observation of a RyR2 involvement during memory processes [15], we hypothesized that the specific neuronal function of RyR2 could provide a crucial link between structural plasticity and memory. To study this link directly, we specifically deleted RyR2 in neurons of the hippocampus, a key region for spatial and contextual memory [53, 54]. Throughout several approaches, we consistently found that depletion of RyR2 resulted in a decreased spine density, reduced activity-dependent spine remodeling and impaired spatial memory acquisition. These findings position RyR2 at a crucial interface between neuronal structure and mnemonic functions.

Using different models, we consistently found that RyR2 function is mechanistically linked to the maintenance of dendritic spines. Both, the pharmacological inhibition of RyRs as well as the downregulation of *Ryr2* reduced the number of dendritic spines. Our findings agree with previous in vitro work in rat primary hippocampal neurons [11, 19, 20]. Here, we now established the in vivo relevance of RyRs function for spine density regulation in the hippocampus. Our findings specifically highlighted the relevance of RyR2, as no alteration of hippocampal spine density could be observed in adult RyR3 knockout mice [21], while the in vivo function of RyR1 remains unknown. Furthermore, we provided electrophysiological evidence supporting that structural spine loss was paralleled by a loss of excitatory synaptic connectivity on a functional level. In addition, in all our approaches dendritic spine loss was consistently more pronounced in distal dendrites, following a pattern observed in several neurological disorders [55, 56], which have in common that they are associated with cellular and network excitability [57, 58]. A possible mechanism underlying the pronounced loss of spines at terminal dendritic branches may be the selective early vulnerability due to an impaired mitochondrial dynamics and transport [59–61]. A possible scenario involves a dysregulation of RyRs-dependent nitric oxide production [62], which was shown previously to alter mitochondrial fission. Altered mitochondrial fission has been associated with the pathogenesis of Alzheimer’s disease [63]. Moreover, in our study RyR2 knockout affected predominantly maintenance of dendritic spines of basal dendrites, which are a key recipient of the commissural input from the contralateral hemisphere [64]. Thus, RyR2 may be linked to synaptic homeostasis required for cross-hemispheric transfer of memory-relevant information [65, 66]. Along these lines, the dendritic structural sparsification that we found in response to RyR2 deletion was reliably observed in both, human neurodegenerative diseases [67–69] and animal models [42, 70–72].

In a previous study, we described that RyR2 is upregulated via an activity-dependent mechanism [23]. We demonstrated that *Ryr2* transcription requires a sustained intracellular signaling cascade involving Ca^2+^-induced Ca^2+^ release, which results in CREB phosphorylation, nuclear re-localization and binding of *Ryr2* promoter region. Consistent with this activity-dependent mechanism, different activating stimuli such as spatial training [11, 24] and the administration of cocaine [23, 73] have been shown to trigger RyR2 upregulation in the hippocampus. Therefore, as a rationale for the current study we used several plasticity-inducing and RyR2 up-regulating paradigms (cLTP induction, spatial learning and cocaine-induced contextual memory) to understand the involvement of RyR2 in hippocampal structural plasticity. Our first observation was in line with previous in vitro work that linked RyRs to the plastic remodeling of spines [11, 19, 20, 74]. We observed that RyR2 function was necessary for cLTP-induced spine plasticity in primary hippocampal neurons. Second, we used spatial learning as a plasticity-inducing paradigm, which led to RyR2 upregulation in the hippocampus as expected [11, 24]. This paradigm effectively stimulated hippocampal spine plasticity. Notably, this effect depended on RyR2 function, confirming an important role of RyR2 for structural plasticity in the brain. Third, we substantiated the relevance of RyR2 for in vivo processes by showing that structural plasticity in response to chronic treatment with the psychoactive drug cocaine was blocked by RyR2 deletion. In agreement with previous studies, we further confirmed that cocaine-treatment resulted in upregulation of RyR2 in the hippocampus [23, 73]. Together, our data support the notion that activity-dependent RyR2 function is a physiological regulator of neuronal long-term structural plasticity, independent of the nature of the stimulus.

Our present work demonstrated clearly, by the acute deletion of RyR2, that RyR2 is required for dendritic spine maintenance in post-developmental brain. This is relevant since developmental RyRs-dependent dendritic Ca^2+^ wave mechanisms of synaptic clustering have been described [36, 75]. Furthermore, this approach opened a window of opportunity for studying putative compensatory mechanisms following RyR2 dysregulation. This was important because, apart from post-translation modifications of RyR2 [26], downregulation of RyR2 expression levels was observed in both, humans samples [28, 39] and an animal model of Alzheimer’s disease [38]. Following acute RyR2 deletion, we observed compensatory changes on two levels. Pyramidal CA1 neurons exhibited increased cellular excitability and altered functional tuning during behavior. At single cell level, we observed higher input resistance and increased neuronal firing rate upon current injection. These findings support the reduction in cell size due to dendritic sparsification. Two phenomena that have been functionally linked previously in physiological conditions [76, 77] and disease models [42, 78]. Both, loss of synapses and an increased neuronal excitability may perturb the functional organization at the circuit level that controls neuronal activity levels during behavior [79]. Thus, we used genetically encoded Ca^2+^ indicators to monitor Ca^2+^ transients as a proxy for neuronal firing in awake animals. We detected a 4-fold increase in the frequency of Ca^2+^ transients in the pyramidal layer of the CA1 of RyR2 knockout mice, which is an in vivo confirmation of the increased cellular excitability that we have observed in the slice preparation. In this direction, several reports support a disrupted neuronal network associated with hyperexcitability and dysfunctional synchronization in animal models and humans affected by Alzheimer’s disease [57, 58]. The most prominent feature of CA1 pyramidal cells is a pronounced spatial representation [80], within the neuronal activity, which we also observed in our Ca^2+^ imaging data (place cells). However, the proportion of place cells within the monitored cell population and the reliability of spatial firing on a single trial was clearly reduced. These findings resembled the disrupted activity pattern of spatially tuned neurons in AD models [49, 81]. It is likely that these altered activity patterns contribute to the impairment of spatial learning and memory, which was previously described [11, 15, 18, 19, 82] and re-confirmed in the present study.

5In summary, our findings highlight the specific function of RyR2 in the maintenance and activity-dependent structural plasticity of dendritic spines in the post-developmental brain. Furthermore, our data underpin the importance of RyR2-associated compensatory mechanisms for neurodegenerative diseases in which RyR2 downregulation has been reliably observed. A compensatory increase in cellular excitability, neuronal hyperactivity, an impaired spatial memory processes are key features of Alzheimer’s disease in which the link between molecular changes, spine remodeling and circuit dysfunction is still incompletely understood.

## Materials and methods

### Animals

Mice were kept in cages of two to four with free access to tap water and food (ssniff® V1534-300) under a 12/12 h light/dark cycle. All experimental procedures were performed in accordance with the institutional animal welfare guidelines and were approved by the State Agency for Nature, Environment and Consumer Protection (LANUV) of North Rhine-Westphalia, Germany. The following lines were obtained from Jackson Laboratory (Bar Harbor, ME): *CMV-Cre*^*tg/wt*^ (B6.C-Tg(CMV-cre)1Cgn/J), *Synapsin-Cre*^*tg/wt*^ (B6.Cg-Tg(Syn1-cre)671Jxm/J), *Camk2α-Cre*^*tg/wt*^ (B6.Cg-Tg(Camk2*α*-cre)T29-1Stl/J) and C57BL/J6. Generation of the target vector, ES transfection, homologous recombination, injection in blastocysts and the selection of heterozygous *Ryr2*^*fl/fl*^ mice were performed at InGenious Targeting Laboratory (2200 Smithtown Ave, Ronkonkoma, NY 11779, United States of America). No animals were excluded from any expderiments unless the presence of technical issues and sample size was chosen to ensure at least 3 biological independent replicates for each experiments.

### Antibodies

The following primary antibodies were used: mouse anti-Ryanodine receptor C34 antibody (abcam, 2868), mouse α-Tubulin (sigma, T9026), goat anti-mouse IgG poly-HRP (Invitrogen, 32230), anti-mouse Alexa Fluor 568-conjugated secondary antibody (Life technologies).

### Primary hippocampal neurons

Primary dissociated hippocampal neurons were prepared using E18-19 embryos from pregnant Sprague Dawley rats. Hippocampi were dissected in cold solution (136 mM NaCl, 5.4 mM KCl, 0.2 mM Na2HPO4, 2mMKH2PO4, 16.7 mM glucose, 20.8 mM sucrose, 0.0012% phenol red, and 10 mM HEPES, pH 7.4), mechanically dissociated and digested with trypsin. Neurons were seeded onto 1 mg/ml poly-L-lysine pre-coated 15 mm coverslips at the optimal cell density with DMEM (Gibco) + 10% Horse Serum. Medium was replaced with Neurobasal Medium (1% B27, antibiotics and glutamine) after 4 h. Neurons were transfected at DIV8 with Ca^2+^ phosphate using the following vectors: SMART vector *Ryr2* (sh-RyR2), promoter mCMV expressing Turbo GFP (V3SR11242-240064983, Dharmacon), SMART vector Non-targeting Control (scramble), promotor mCMV expressing Turbo GFP (VSC11708, Dharmacon). To inhibit RyRs, DIV14 neurons transfected with scramble vector were either treated with 50 μM Ryanodine (Tocris) or DMSO for 60 min. To induce cLTP, DIV14 neurons transfected with scramble or sh-RyR2 were incubated in ACSF (125 mM NaCl, 2.5 mM KCl, 1 mM MgCl_2_, 2 mM CaCl_2_, 33 mM D-glucose and 25 mM HEPES, pH 7.3) for 30 min, stimulated in ACSF (without MgCl_2_) with 0.05 mM Forskolin (Sigma Aldrich), 0.1 mM Picrotoxin (Tocris) and 100 nM Rolipram (Calbiochem) for 16 min. Cells were incubated again in ACSF for 44 min. Untreated cells were incubated only in ACSF. After treatments cells were fixed 5 min in 4% paraformaldehyde plus 4% sucrose in PBS.

### Tissue preparation

Animals of 4–6 months of age were sacrificed through cervical dislocation and brain samples were removed, dissected, snap frozen in liquid nitrogen and stored at −80 °C. Mice were alternatively anaesthetized with a mix 1:1 of Ketamine and Xylazine and transcardially perfused with 4% PFA (paraformaldehyde). Brains were further fixed in 4% PFA overnight at 4 °C. The day after, brains were moved to a 30% sucrose solution. Brains were sagittal or coronal sectioned in different series with a thickness between 35–40 μm using the Cryostar NX70 (Thermo Fisher Scientific). One of the series was directly mounted and Nissl-stained as follows using the following steps: EtOH 100% (2 min), EtOH 90% (2 min), EtOH 70% (2 min), bi-distilled water (2 min), Crystal Violet (15 min), bi-distilled water (2 min), EtOH 70 (2 min), EtOH 90 (2 min), EtOH 100 (2 min), Xylol (2 min). Sections were mounted using DePex (VWR) and dried overnight. The other series were frozen at −20 °C in cryoprotective solution.

### Immunostaining

Cell cultures were blocked for 1 h at room temperature in 10% normal goat serum solution and 0.1% Triton-X-100. Primary antibodies were incubated overnight at 4 °C, cells were washed in PBS and incubated with Alexa Fluor-conjugated secondary antibody for 2 h at room temperature. After counterstaining with Hoechst-33342, cells were mounted and coverslipped with fluorescence mounting medium (DAKO).

### In situ hybridization

In situ hybridization was performed using the RNAscope® Multiplex Flourescent Reagent Kit v2 (ACD, a bio-techne brand). Procedures followed the described company’s protocol. Brain sections of 30–35 μm of thickness were mounted on glass slides, dried and permeabilized with RNAscope® Hydrogen Peroxide for 10 min at room temperature. Target antigen retrieval was performed for 5 min at 90 °C. Sections were washed, dehydrated in alcohol and dried at 37 °C. The probe was hybridized and amplified following company’s instructions. TSA® Plus Cyanine 3 (CyR3,1:750) (Perkinelmer) fluorophore was used to detect the amplified probe. After counterstaining with Hoechst-33342, sections were mounted and coverslipped with fluorescence mounting medium (DAKO). The *Ryr2* probe (Cat No. 479981) was purchased from ACD probes to detect the mRNA target region 735–1636 bp.

### Golgi staining

Only male mice of 4–6 months of age were used for the Golgi staining. FD rapid Golgistain_TM_ Kit (FD NeuroTechnologies) was applied following the company’s instructions. Briefly, brains were immersed in a 1:1 mixture of solution A and B for 2 weeks and kept in the dark at room temperature. Solution A + B was replaced after the first 24 h. Brains were moved to Solution C for 120 h at room temperature. Solution C was replaced after the first 24 h. Brains were frozen in methylbutane for 20 s and kept at −80 °C. Brains were sectioned via sagittal cut with a thickness of 100 μm using the cryostat NX70 (Thermo Fisher Scientific). Sections were dried overnight at room temperature and then developed using the following steps: 2 times 4 min in bi-distilled water, 10 min in the development solution (1:1:2 of solution D:E:bi-distilled water), 2 times 4 min in bi-distilled water, 4 min in EtOH 50%, EtOH 70%, EtOH 90%, 3 times in EtOH 100% and a final step in Xylol. Sections were mounted using DePex (VWR) and dried overnight.

### Imaging and neuronal 3D reconstructions

Brain sections and cells stained with fluorescent dyes were imaged with the LSM 700 Zeiss confocal microscope equipped with ZEN 2012 black edition (Zeiss) and with 20x, 40x, 63x Plan-Apochromat objectives. Nissl- and Golgi-stained slices were imaged using Zeiss EPI-SCOPE1 Apotome (20x, 40x and 63x objectives) in bright field. The microscope was equipped with ZEN 2012 blue edition (Zeiss). Spine density in golgi-stained sections was manually counted by a trained examiner blinded to treatment conditions using ImageJ (US National Institutes of Health, Maryland, USA) and z-stack of 10–50 planes with a pixel dimension of *x* = 0.073 μm *y = 0.073 μm *z = 0.28 μm. Neurons filled with biocytin (0.4%) were reconstructed in 3D taking z-stacks of 70–130 planes imaged using a 20x objective. Neurons were analyzed with Imaris FilamentTracer (Bitplane). After loading z-stacks in Imaris “arena”, a semiautomatic reconstruction was done with different detection methods: “AutoPath” and “AutoDepth”. FilamentTracer provided interactive measurements used to quantify different neuronal parameters: dendrite volume, dendrite area, dendrite length, dendrite thickness and number of dendrite branches. This feature required Imaris MeasurementPro. Neurons filled with biocytin (0.4%) were also used to reconstruct dendritic spine in 3D. Z-stacks of 10–50 planes were imaged with a 63x objective. Prior the reconstruction, images were deconvoluted using Huygens software (Scientific Volume Imaging). NeuronStudio was finally used for the semi-automatic morphological quantification of spine [83, 84].

### Behavioral tests

Male and female mice were used for the behavioral assessments. Prior to behavioral testing, mice were handled daily for seven days. Testing started when the mice were between 12 and 16 weeks old. To minimize the effects of previous tests on subsequent behavior, we performed the behavioral test battery in a specific order in which the less stressful tests preceded the more stressful ones. The individual behavioral tests were blinded and separated by one week. *Open field*. Spontaneous locomotor activity and exploratory behavior was assessed in open field boxes (27 cm × 27 cm × 27 cm) in an evenly lit room. Each mouse was placed individually in the center of the box and its behavior was recorded for 20 min. The distance moved (cm) as well as the velocity (cm/s) were analyzed using the EthoVision tracking system (Noldus, The Netherlands). *Rotarod*. Motor coordination was assessed using a rotarod system (TSE Systems, Bad Homburg, Germany). Mice were trained to remain on the rod, which rotated with accelerating speed (4–40 rpm), over three consecutive days with three daily sessions and a maximum session duration of 5 min. A 30 min break was given between individual training sessions. When a mouse fell from the rod, infrared light beams at the bottom of the chamber were interrupted allowing measuring the fall latency (s). *Y-maze*. The Y-maze apparatus was used to evaluate short-term spatial memory function. It consisted of three equilateral arms made of plastic. The maze was placed in a well-lit room containing several external cues. During the first trial, one of the arms was blocked with an opaque door. Each mouse was placed in the end of the start arm and allowed to explore the two open arms for 10 min. Then, it was removed and returned to its home cage. Following a delay of 90 min, a test trial was performed during which the previously closed arm was freely accessible. The mouse was reintroduced into the maze and the number of arm entries as well as the time spent in each arm was recorded for 5 min using the EthoVision tracking system (Noldus, The Netherlands). *Spontaneous alternation*. Working memory function was assessed by measuring spontaneous alternation behavior in the y-maze. Mice were subjected to a three-arm y-maze (dimensions described above) for 6 min with all arms open. The number and sequence of arms entered were scored by an observer blind to genotype group. The dependent variables were activity, defined as the number of arms entered, and percentage alternation, calculated by the number of alternations (consecutive entries into three different arms) divided by the number of total possible alternations (i.e., the number of arms entered minus 2) and multiplied by 100. *Morris water maze*. The Morris water maze (MWM) is a commonly used behavioral test to assess hippocampus-dependent spatial learning and memory in rodents. Here, the test apparatus consisted of a white circular swimming pool (150 cm in diameter) filled with water that was maintained at 22 °C and made opaque with white paint. The maze was divided into four quadrants (target, right, opposite, left). A transparent Plexiglas platform was positioned in a fixed location in the pool and submerged 0.5 cm below the water surface. Mice were trained to locate the platform position over five consecutive days with four to six trials per day and a maximum trial duration of 60 s. The starting positions were semi-randomized so that each trial started from a different quadrant. Short-term memory was tested in a 60 s probe trial 90 min after the last block of training trials on day 3, while long-term memory was assessed on day 6, 24 h after the last training session. During training sessions, the latency to find the platform (escape latency, s), the swim speed (cm/s) as well as the swim distance (cm) were analyzed using a video tracking system (EthoVision). Memory performance during probe trials was assessed via the time spent in the target compared to non-target quadrants (quadrant occupancy) as well as the number of target crossings. Mice were sacrificed 24 h after the Long-term Memory test. *Contextual fear conditioning*. Fear conditioning was conducted in transparent plastic boxes (21.5 cm × 20 cm × 25 cm) with stainless-steel grid floors connected to an aversive stimulator (Med Associates). For fear conditioning, mice were placed individually into the chamber and allowed to habituate for 2 min. Thereafter, three shocks (0.75 mA, 2 s) with an inter-shock interval of 60 s were delivered. Animals were removed from the boxes 60 s after the last shock and placed back into their home cages. In order to examine memory retrieval, mice were re-introduced into the chambers 24 h after the conditioning procedure for 5 min without the presentation of foot shocks. During fear conditioning and retrieval, mice were video recorded and the time spent freezing was analyzed. *Conditioned place preference test*. Place conditioning was performed as described previously. Briefly, animals were handled for 5 min one day prior to the experiment (day 1). Baseline preferences for either the black or white compartment of the CPP box were assessed during the pre-test (day 2). Therefore, mice were placed individually in the center compartment of the three-chamber place preference apparatus with the guillotine doors open, allowing free access to all three distinct compartments for 20 min. The time spent in each compartment was recorded using a video tracking system (EthoVision). Conditioning was conducted over the next 6 consecutive days (day 3–day 8) with the guillotine doors closed, thus confining the mice to a specific chamber for 20 min following the injection of either saline or cocaine (20 mg/kg, i.p.). Using a pseudo-randomized approach, half the animals were placed into the black compartment following cocaine injection while the other half was placed into the white compartment after drug administration. On the next day, mice were injected with 0.9% saline before placement into the alternate chamber. Thereafter, injections were alternated for the subsequent 4 days of the conditioning phase. Twenty-four hours after the last conditioning session (i.e., saline administration), cocaine-induced place preference was assessed for 20 min by placing the animals into the center compartment with all guillotine doors open. The CPP score was calculated as the time (s) spent in cocaine-paired minus the time spent in the saline –paired compartment. In order to identify cocaine-induced changes in spine density, one cohort of *Camk2α-Cre*^*tg/wt*^*;Ryr2*^*fl/fl*^ and control mice received only saline injections over the entire conditioning phase before being placed in either the white or black chamber of the CPP box. Mice were sacrificed 24 h after the CPP test. *Radial arm maze*. Animals were habituated to the reward one week before the start of the experiment. For this purpose, animals were exposed to the reward (peanut butter) for 30 min inside the home cage. Three days preceding the start of the experiment, animals were habituated to procedure. Inside the home cage, animals were transported to experimental room. Then each animal was carefully lifted up and kept in the hand for around 30 s. Afterward, the just lifted animal was placed in a separated cage. This was repeated five times. The whole procedure was performed twice a day (in the morning and in the afternoon). During the first experimental day, each animal was allowed to familiarize with the arena. For this purpose, animals were placed inside the experimental arena for 10 min so that they were able to explore it freely. At this point, there were no baits installed at the end of the arms. Testing was then performed on the following 5 consecutive days. Animals were allowed to search for the peanut butter reward for 10 min. If the baits had been cleared before the end of the 10 min testing period, animals were removed from the arena after consumption of the last reward. After testing, animal was placed not in the home cage but in a different, separated cage. Animals were always tested at around the same time of the day. Errors were defined as arm-entries without consumption of baits.

### Electrophysiology and reconstruction of neurons

*AAV-injection*. Stereotactic viral injections were performed as described previously in detail [45]. In brief, 5-months old male mice were anesthetized with i.p. injection of Ketamine (0.13 mg/g) and Xylazine (0.01 mg/g); head-fixed using a head holder (MA-6N, Narishige, Tokyo, Japan) and placed into a motorized stereotactic frame (Luigs-Neumann, Ratingen, Germany). Body temperature was constantly controlled by a self-regulating heating pad (Fine Science Tools, Heidelberg, Germany). After skin incision and removal of the pericranium, position of the injection 34 G cannula was determined in relation to bregma. A 0.5 mm hole was drilled through the skull (Ideal micro drill, World Precision Instruments, Berlin, Germany). Stereotactic coordinates were taken from Franklin and Paxinos, 2008 (The Mouse Brain in Stereotaxic Coordinates, Third Edition, Academic Press). Virus was injected in two loci with the following coordinates: anterior-posterior: −2(−2,5 mm), lateral −2,3(−3 mm) and ventral −1,4(−1,8 mm) relative to Bregma into the right hippocampus under the 10 °C angle at 0.1 ml/min using a UltraMicroPump with Hamilton syringe inserted (World Precision Instruments, Berlin, Germany). Two virus were injected in the separate groups of mice *rAAV5.CamK2α-GFP-cre* (Gene Therapy Center Vector Core, The University of North Carolina at Chapel Hill) (*n* = 15) and *AAV5.CamK2α-GFP* as a control vector (UNC, Gene Therapy, Center VectorCore) (*n* = 15). Electrophysiological experiments were performed 4–6 weeks after virus injection. In vitro patch-clamp recording in horizontal hippocampal slices. Only male mice were used for the patch-clamp recording. Experiments were performed blinded in transverse hippocampal slices (thickness: 300 µM) [45], using a VT-1200S vibratome (Leica Microsystems, Wetzlar, Germany) in ice-cold sucrose solution containing (mM): 60 NaCl, 100 sucrose, 2.5 KCl, 1.25 NaH_2_PO_4_, 26 NaHCO_3_, 1 CaCl_2_, 5 MgCl_2_, 20 glucose, oxygenated with 95% O_2_ and 5% CO_2_. Hippocampal transverse slices were placed for 30 min at 35 °C for recovery and replaced into standard ACSF with the following composition (mM): 125 NaCl, 3 KCl, 1.25 NaH_2_PO_4_, 26 NaHCO_3_, 2.6 CaCl_2_, 1.3 MgCl_2_, 15 glucose) at room temperature, where they were kept up to 6 h for patch-clamp recording. Whole-cell patch-clamp recordings were obtained from neurons in CA1 pyramidal neurons visualized by infrared DIC or oblique illumination. After confirmation of presence of GFP, neurons were recorded either in current-clamp mode using a BVC-700A amplifier (Dagan, Minneapolis, USA) or in voltage-clamp mode using an Axopatch 200B amplifier (Molecular Devices, LLC, Sunnyvale, CA, USA). Signals were digitalized at 50 kHz or higher sampling rates using an ITC-18 interface board (HEKA Elektronik, Lambrecht, Germany) controlled by IgorPro 6.7 software (WaveMetrics, Portland, USA). The recording pipettes with a resistance of 3–5 MΩ were filled with intracellular solution containing (mM): 140 K-gluconate, 7 KCl, 5 HEPES-acid, 0.5 MgCl_2_, 5 phosphocreatine, implemented with biocytin (0.4%) (adjusted to pH 7.4 with KOH). All recordings were performed at 34 °C. Cells with a resting membrane potential higher than −50 mV were excluded. Recordings were stopped, when the series resistance exceeded 40 MΩ. Intrinsic properties of the neurons were estimated at the membrane potential −60 mV with current injection in depolarization and hyperpolarization direction with following amplitude: −200 pA, −30 pA, −20 pA, −10 pA, 0 pA, +10 pA, +20 pA, +30 pA, +200 pA. Resting membrane potential was determined immediately after establishing whole-cell configuration. Input resistance was calculated from linear fitting the range of current injections (−30 pA, −20 pA, −10 pA and 0 pA). Half-width of the first and second action potential, as well as initial firing frequency, were estimated at +200 pA current injection, sag ratio at −200 pA. *Miniature EPSCs measurements*. For miniature excitatory postsynaptic current (mEPSC) measurements TTX (1 μM) was bath applied. In addition, GABAergic synaptic transmission was blocked with Gabazine (SR-95531, 10 μM) and CGP 52432 (1 μM; TOCRIS Bioscience, Bristol, UK). Cells were kept at a holding potential of −80 mV after correction of the liquid junction potential (10.5 mV). Cell capacitance was canceled and series resistance was not compensated, but carefully monitored, in accordance to established protocols [85]. Recordings were stopped, when the series resistance exceeded 30 MΩ. Only continuous recordings longer than 20 min were processed. Potential events were extracted from the recordings by matching of the following function [86] to the single events:

$$f\left( t \right) = scale\left( {1 - e^{ - {\textstyle{t \over {\tau _{_{on}}}}}}} \right)^5\left( {e^{ - {\textstyle{t \over {\tau _{_{on}}}}}}} \right)$$

Events were classified as mEPSCs when their amplitude excided two times the baseline standard deviation together with the temporal parameters to discard false events. *Morphological reconstruction of neurons and spines*. Neurons were reconstructed after electrophysiological recording by a blinded operator. Slices were fixed over-night in paraformaldehyde (4%), washed 2 times with 0.1 M PBS at room temperature, incubated 15 min in 0.5% Triton X-100, stained using streptavidin-coupled Alexa 555 (4 mg/ml in TBS-2%NGS) (Life technologies, S21381) for 2 h and washed 2 times 15 min in PBS. Slices were mounted with Poly Aqua Mount (Polysciences) and stored at 4 °C in the dark.

### Two-photon in vivo Ca^2+^ experiments

AAV-injection and chronic hippocampal window surgery for in vivo Ca^2+^ imaging. Stereotactic viral injections for in vivo Ca^2+^ imaging were performed as described before [43, 44]. Surgery was performed 3 months before start of the imaging experiments. At the time of the surgery the male mice were between 5–7 months old. Mice were anaesthetized using an intraperitoneal (i.p.) injection of ketamine (0.13 mg/g) and xylazine (0.01 mg/g bodyweight). In addition, preceding the surgery the animals receive a subcutaneous (s.c.) dose of buprenorphine (0.05 mg/kg). The eyes were covered with eye ointment (Bepanthen, Bayer, Germany) to prevent drying. During the surgery, the mice were placed on a heating blanket to maintain the body temperature. The depth of the anesthesia and the analgesia was tested by pinching the toe and checking for the absence of the toe-pinch withdrawal reflex. The animals were fixed inside a stereotactic frame and the skin at the site of the surgery was disinfected using 70% ethanol. A small incision into the skin along the sutura sagittalis was made which allowed for lifting the skin and shifting aside the periosteum. Small craniotomies (~0.5–1 mm diameter) were drilled at −1.9 mm anteroposterior (AP) and ±1.25 mm mediolateral (ML) relative to bregma. The injection needle was lowered to a depth of −1 mm dorsoventral from brain surface and a total volume of 1 µL of the adeno-associated virus (AAV) was injected into the CA1 region of the hippocampus at a speed of 100 nL/min. For imaging experiments, *Ryr2*^fl/fl^ mice were injected with 0.500 µL of *AAV9.CamK2α0.4.Cre.SV40* (1 × 10^13^ vg/mL, Addgene) and 0.500 µL of pAAV1.Syn.Flex.GCaMP6m.WPRE.SV40 (1 × 10^13^ vg/mL, Addgene). C57BL/6 controls were injected with equal amounts of the same AAV-combination. For behavioral experiments, *Ryr2*^fl/fl^ controls were injected with 1 µL of *pAAV.Syn.GCaMP6m.WPRE.SV40* (1 × 10^13^ vg/mL, Addgene). The needle was left in place for 5 min in order to allow diffusion of the AAV and then carefully removed. The same procedure was repeated for the contralateral injection site. Afterwards, the wound was stitched and disinfected using povidone-iodine (Betaisodona). During the next three days the mice were monitored and subcutaneously treated with buprenorphine (0.05 mg/kg) every 8 h. Implantation of the chronic hippocampal window was performed one week after AAV injection. Mice were anesthetized as already described for the AAV injection. In addition, dexamethasone (0.2 mg/kg) was applied s.c. to reduce swelling and inflammation of the tissue. Eye-ointment was applied to the eyes and body temperature was maintained at 37 °C. The depth of anesthesia and analgesia was tested and when sufficient depth was reached, the animal was fixed to a stereotactic frame. The skin above the skull was disinfected with 70% ethanol. Next, the skin above the skull was removed and the exposed cranial bone was cleaned and dried with Sugi absorbant swabs (Kettenbach GmbH, Germany). The surface of the bone was scraped and cleaned with a scalpel. For preparing a base-layer of glue to which the metal bar was subsequently fixed, we used a two-component dental glue (OptiBond, FL, Kerr, USA). After drying and roughening of the skull, a thin layer of the priming component was applied for 15 s to the skull surface with a slight scrubbing motion. The layer was gently air-dried for 5 s. Using the same applicator-brush, a layer of the adhesive component was applied to the skull surface the same way as it was done with the priming component. The glue was the light cured for 40 s. Using a dental drill, a circular piece (Ø 3 mm) of the skull above the right somatosensory cortex (AP: −2.2, ML: −1.8 relative to bregma) was removed. Then the dura was removed using a very fine forceps and the somatosensory cortex was aspirated with a 27-gauge needle attached to a syringe, a flexible tube and a vacuum pump. The surgery site was rinsed with sterile PBS (pH 7.4) over the entire time of the process. When the cortex tissue lying above the dorsal hippocampus was removed entirely, the external capsule was cautiously peeled away until the alveus was exposed. The surgery site was rinsed with sterile PBS (pH 7.4) until bleeding stopped. Now, a metal tube (Ø: 3 mm, h: 1.5 mm) sealed with a glass cover-slip (Ø: 3 mm, h: 0.17 mm) was inserted and the upper tube edge was glued to the skull bone using super glue. When the super glue was hardened, dental cement was used to fix a metal bar to the contralateral side of the window. After surgery, the animal received a dose of buprenorphine (0.05 mg/kg) and was allowed to wake up from anesthesia in an incubation box set to 25 °C. The animal was subsequently monitored and treated with buprenorphine (0.05 mg/kg) every 8 h for 3 days. Animal training for in vivo Ca^2+^ imaging. Animal training started approximately 2 months after AAV injection. First, the animals were habituated to the experimenter twice a day for 5–7 days. The habituation period was defined as completed when the animal was voluntarily climbing on the outstretched hand of the experimenter. Next, the animals were familiarized with the reward solution (20% sucrose). Before start of the imaging experiment, the animals were fixed on the linear track for 1 h for 3–5 consecutive days until they were running 20 laps in 1 h. Two-photon in vivo Ca^2+^ imaging. For in-vivo Ca^2+^ imaging, a small metal adapter was attached to the headbar already fixed to the head of the animal. The animals were transferred to a stereotactic frame that fixed the animal on the linear treadmill. For movement recording of the animal, we used a camera (Sumikon PX-8262-919) and an optical mouse detecting the position of the animal on the belt. The camera was turned on manually. The recording of the linear treadmill position was triggered by the start of the scanner and controlled using a custom-written Python 2.7 script. For the alignment of the different hardware components we used an ITC-18 board (HEKA Instruments Inc., Bellmore, USA) and the IGOR Pro software (WaveMetrics). The reward was delivered at every turn of the belt through a custom-built application system consisting of a pump and a blunt 21gauge needle. Time-laps recordings of Ca^2+^-changes were performed with a galvo-resonant scanner (Thorlabs, Newton, USA) on a two-photon microscope equipped with a 16x objective (N16XLWD-PF, Nikon, Düsseldorf, Germany) and a Ti:Sa laser that was tuned to 920 nm for GCaMP6m fluorescence excitation. GCaMP6m fluorescence emission was detected using a band-pass filter (525/50, AHF, Tübingen, Germany) and a GaAsP PMT (Thorlabs, Newton, USA). ThorImageLS software (Thorlabs, version 2.1) was used to control image acquisition. Image series (896 × 480 pixels, 0.715 µm/pixel) were acquired at 32.3 Hz. Imaging data processing, segmentation and data extraction. Motion correction of time-series data was performed using a combination of efficient subpixel registration and in-frame motion correction with the Lucas-Kanade method. Data that showed strong motion artefacts even after registration were discarded. For extracting the fluorescence signal over time from principle CA1 neurons for the Ca^2+^ event frequency analysis, one minute periods where the animal showed no movement were selected and an average intensity projection was created from the processed imaging data. Regions-of-interests (ROIs) were drawn manually around single-cell bodies using ImageJ/Fiji. The change in mean gray value over time was extracted for each ROI. Analysis of Ca^2+^ event frequency. First, the ΔF/F was calculated by normalizing each single raw Ca^2+^ trace derived from the ROIs to the lowest 20% of all fluorescence intensity values of that same trace. In order to get a measure of neuronal activity we inferred the underlying spiking activity from the ΔF/F traces. We used the OASIS module for Ca^2+^ deconvolution [46] that is implemented in the freely available CaImAn toolbox [87]. The algorithm we applied is using the threshold non-negative least square (NNLS) method with a deconvolution kernel. The kernel is modeled as the difference between two exponential functions:

$$h\left( t \right) = \frac{{\left(e^{\frac{{ - t}}{{\tau _d}}} - e^{\frac{{ - t}}{{\tau _r}}}\right)}}{{\tau _d - \tau _r}}$$

where τ_d_ and τ_r_ are the roots of the polynomial:

$${\mathrm{f}}\left( {\mathrm{x}} \right) = {\mathrm{x}}^2 \, {-} \, 1.7{\mathrm{x}} + 0.712$$

The algorithm extracts a denoised trace from the original ΔF/F traces where the peaks in the original trace are fitted to the deconvolution kernel. All data points that are not part of an identified peak are set to 0. The deconvolved trace typically has values of varying size in neighboring bins which reflects the uncertainty regarding the exact position of the spike. Also in very noisy traces this can lead to noise being recognized as “partial spike”. The algorithm has implemented a constraint on the minimal spike size s_min_ which we set in dependency of the calculated spectral noise (sn).

$${\mathrm{s}}_{{\mathrm{min}}} = {\mathrm{sn}} \ast 2$$

By doing this we correct for the difference in signal-to-noise ratio of the recordings. A higher s_min_ value would require a Ca^2+^ event to be more prominent relative to baseline in order to be detected as spike. In turn, a small s_min_ value already would allow Ca^2+^ events that have relatively small amplitude to get detected as spikes. In an additional step, we set another threshold for the now detected spikes which allowed us to filter out some additional false-positive spikes. Analysis of spatially tuned firing. The analysis was performed using ImageJ and custom scripts written in python (version 2.7). Time series datasets were motion-corrected as previously described. ROIs were manually drawn around cells that showed visible Ca^2+^ events during phases where the animal was running. From these ROIs, raw Ca^2+^ traces were extracted for those periods where the animal reached a velocity of 3 cm s^−1^. The ΔF/F was calculated by normalizing the single raw Ca^2+^ trace derived from the ROIs to the lowest 20% of all fluorescence intensity values of that same trace. The identification of place cells from Ca^2+^ activity is based on a slightly adjusted procedure [88]. For this in the first step the ΔF/F signal was aligned with the respective position on the linear track. For this, the track (in total 2 m of length) was separated into spatial bins of 2 cm yielding 100 different spatial bins. For each cell, the ΔF/F was calculated as a function of virtual position for the 100 positions. Potential place cells were first identified as contiguous regions of this plot in which all of the points were greater than 25% of the difference between the peak ΔF/F value (for all 100 bins) and the baseline value (mean of lowest 25 out of 100 ΔF/F values). These identified potential place fields had to satisfy following criteria: (i) The minimal length of the place field has to be >16 cm (or 8 bins). (ii) At least one value has to be 10% mean ΔF/F. (iii) The mean in-field ΔF/F has to be more than two times the out-field ΔF/F. (iv) A significant transient has to be present in at least 33% of the times the animal passes through the place field. A significant transient was defined as a ΔF/F transient that lead to four ΔF/F values being larger than 2.5 times the rolling mean plus the rolling standard deviation in a window of around 6 s.

### Southern blot

Genetic deletion of *Ryr2* loci was confirmed via Southern blot analysis. Briefly, genomic DNA (gDNA) from tissues was isolated using standard isopropanol/ethanol isolation procedure, with minor modifications. gDNA was digested, electrophoretically separated on an agarose gel and blotted onto Amersham Hybond-XL nylon membrane (GE Healthcare). After blotting, membranes were rinsed in 2X SSC buffer (AppliChem) and DNA fixed via incubation at 80 °C. The Southern blot probe used for the hybridization was amplified with Polymerase chain reaction (PCR) using Taq DNA Polymerase recombinant (Life Technologies), with the primers 5′-GACTGAAACAGGGTGCGCTC-3′ and 5′-CCGCTGATGGATAATGAAACTCC-3′. Primers for the amplification were designed by the iTL company. The obtained fragment was electrophoretically separated on agarose to verify correct size and the 10049 bp amplicon purified using QIAquick® Gel Extraction Kit (QIAGEN), following manufacturer’s instruction. The probe was radioactively labeled with α-[32P]-dCTP, using RediprimeTMII Random-Prime Labeling System-Kit (Amersham) and unincorporated nucleotides removed using illustra ProbeQuant G-50 Micro Column (GE Healthcare), following manufacturer’s instruction. The membrane was incubated with the radioactively labeled probe ON at 65 °C. Fragment size was visualized using HyperfilmTM MP (GE Healthcare): labeled membranes were incubated in an autoradiography chamber at −80 °C for 3 days and films developed using a CURIX 60 (AGFA) automated developing machine.

### Real-time PCR

RT-PCR was used to quantify gene expression levels. RNA was extracted from snap frozen brains using RNeasy kit (Qiagen) and tested for purify (A260/280) and integrity. RNA extracted (100 ng) was retrotranscribed with qScript cDNA SuperMix (Quanta Biosciences). Gene expression analysis was conducted with Fast SYBR Green Master Mix (Applied Biosystems) on a Step One Plus Real-Time PCR System (Applied Biosystems). The following primers were used: *Ryr1* F 5′-AGCTGACAATAAGAGCAAAATGG-3′; *Ryr1* R 5′-TGATCTGAGCCACCTGACTG-3′; *Ryr2* F 5′-CTACCCGAACCTCCAGCGATACT-3′; *Ryr2* R 5′-GCAAAAGAAGGAGATGATGGTGTG-3′; *Ryr3* F 5′-CAGTGGGTATGCTTCCCATAA-3′; *Ryr3* R 5′-GGCCAGCTTGCAGAATAGG-3′; *Ip3r1* F 5′-CCACAGAGCAGGAGCTTGAA-3′; *Ip3r1* R 5′-TTGCCAAAGCTGGTAAGGCT-3′; *Ip3r2* F 5′-ACATCGTGTCCCTGTACGC-3′; *Ip3r2* R 5′-TGGCTGCAAAGAGGTCAACT-3′; *Ip3r3* F 5′-CTACCCGAACCTCCAGCGATACT-3′; *Ip3r3* 5′-CAGGAACTTCTCCTGCTCCG-3′; *Serca2* F 5′-GATGGGGCTCCAACGAATTG-3′; *Serca2* R 5′-TCTTCCCCTTCCTCGAACCA-3′; *Serca3* F 5′-GACGCTCACCACCAATCAGA-3′; *Serca3* R 5′-CTCCCCTTGCCTCACTTCG-3′; *Ca*_*V*_ *1.2* F 5′-CCTGGCCATGCAGCACTAT-3′; *Ca*_*V*_ *1.2* R 5′-GCTCCCAATGACGATGAGGA-3′; *Ca*_*V*_ *1.3* F 5′-TGGCCATGCAGCACTATGAG-3′; *Ca*_*V*_ *1.3* R 5′- CGTGTTCCAGGCGTCACTAA-3′; ΔΔCt values were normilized to β-actin and represented as fold change compared to control mice.

### SDS gel electrophoresis and western blot analysis

Samples were prepared homogenizing frozen tissues in RIPA buffer (Sigma Aldrich) supplemented with proteases (complete, ETDA-free, Roche) and phosphatases inhibitor (PhospoSTOP, Roche) using an IKA Ultra-Turrax homogenizer in ice. Proteins extracted were subjected to SDS-page and immunoblot on nitrocellulose membranes (Amersham). Between 20 and 40 μg of protein were loaded and resolved on 10–12% acrylamide gel and transferred onto nitrocellulose membranes (Bio-Rad). Due to the high molecular weight of RyR2, 7% acrylamide gels were used and stacking gels were kept intact. Overnight transferring on nitrocellulose membranes were run at 20 V for 15 h at 4 °C. Membranes were blocked in 5% milk 0.05% TBS-Tween−20 for 1 h, incubated with the primary antibodies overnight at 4 °C and finally incubated with secondary antibodies for 1 h at room temperature. Immunoblots were developed with SuperSignal West Pico Chemiluminescent Substrate (Thermo-Scientific) and imaged with the chemiluminescent analyzer Chemidoc imaging system (Bio-Rad). Bands were quantified by densitometry (ImageLab software, Bio-Rad).

### Statistics

GraphPad Prism Software (GraphPad Software) was used for statistical analyses. Data are expressed as mean ± SEM or median [25th and 75th percentile]. Number of biological replicates is indicated in the figure legends. Data was tested for normality with the D’Agostino & Pearson normality test. Statistical comparison of normal distributed data was performed with two-tailed Student’s *t* test, one-way ANOVA, RM one-way ANOVA or Pearson correlation. Non-normal distributed data was statistically compared with Mann–Whitney *U*-Test, Friedmann-Test, or Spearman Correlation, respectively. Statistical significance was defined as ^∗^*p* < 0.05.

## Supplementary information

Supplementary Figure S1

Supplementary Figure S2

Supplementary Figure S3

Supplementary Figure S4

Supplementary Figure S5

Supplementary Figure S6

Table S1

Table S2.

Supplementary Figure Legends
