# Correction: Loss of Ryanodine Receptor 2 impairs neuronal activity-dependent remodeling of dendritic spines and triggers compensatory neuronal hyperexcitability

**DOI:** 10.1038/s41418-020-00605-x

**Published:** 2020-08-19

**Authors:** Fabio Bertan, Lena Wischhof, Liudmila Sosulina, Manuel Mittag, Dennis Dalügge, Alessandra Fornarelli, Fabrizio Gardoni, Elena Marcello, Monica Di Luca, Martin Fuhrmann, Stefan Remy, Daniele Bano, Pierluigi Nicotera

**Affiliations:** 1 German Center for Neurodegenerative Diseases (DZNE), Bonn, Germany; 2 Department of Pharmacological and Biomolecular Sciences, Università degli Studi di Milano, Milan, Italy; 3 Department of Cellular Neuroscience, Leibniz Institute for Neurobiology, Magdeburg, Germany

**Keywords:** Neuroscience, Neurological disorders

Correction to: *Cell Death & Differentiation*

10.1038/s41418-020-0584-2 published online 08 July 2020

This Article was originally published under Nature Research’s License to Publish, but has now been made available under a [CC BY 4.0] license. The PDF and HTML versions of the Article have been modified accordingly.

