# Supplementary figures and images for: Loss of Ryanodine Receptor 2 impairs neuronal activity-dependent remodeling of dendritic spines and triggers compensatory neuronal hyperexcitability
Source: Cell Death Differ. 2020 Jul 8;27(12):3354–73. doi: 10.1038/s41418-020-0584-2 (PMC7853040; doi:10.1038/s41418-020-0584-2)

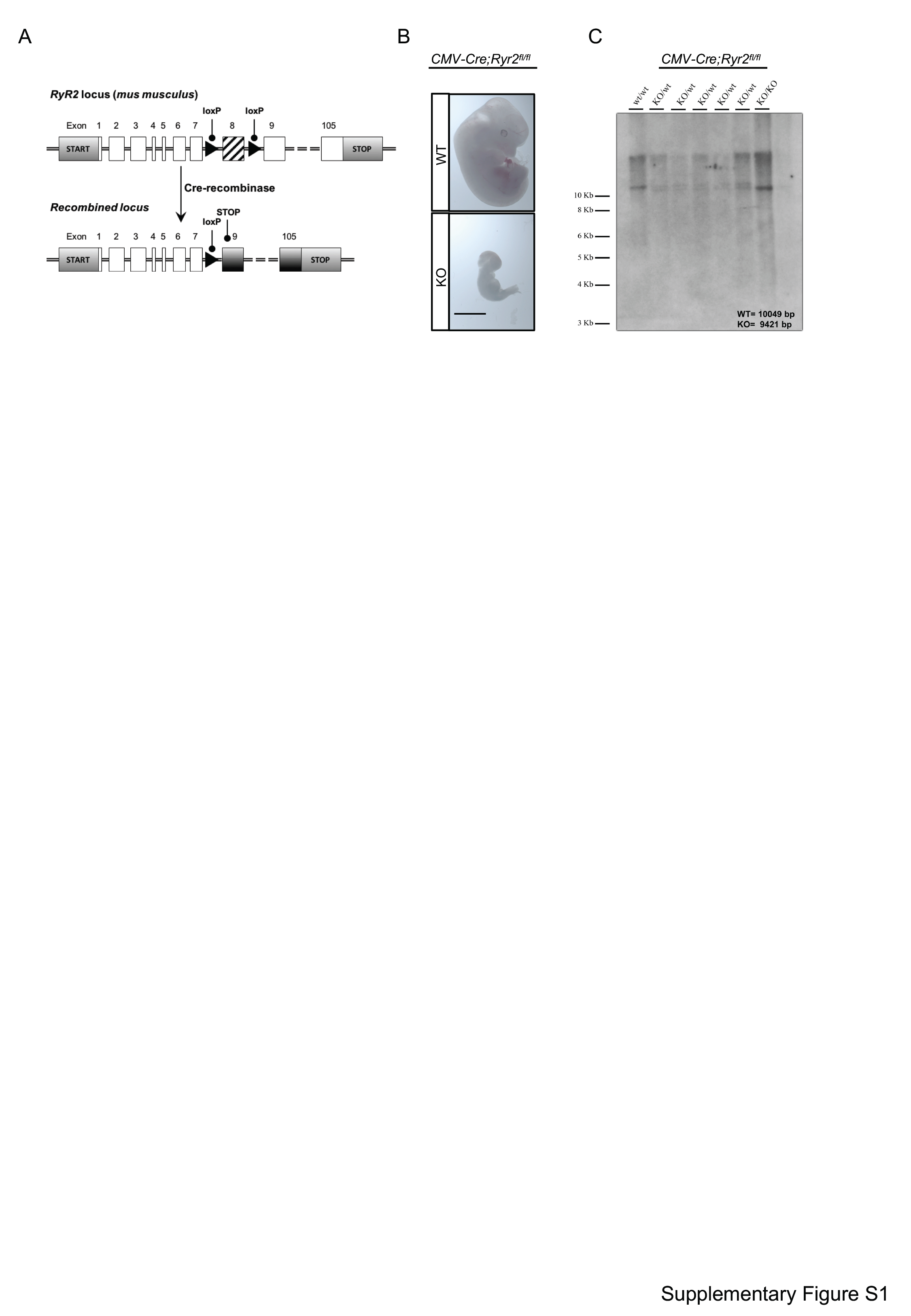

Supplement: Supplementary file 1 — Supplementary Figure S1 [file 41418_2020_584_MOESM1_ESM.png]

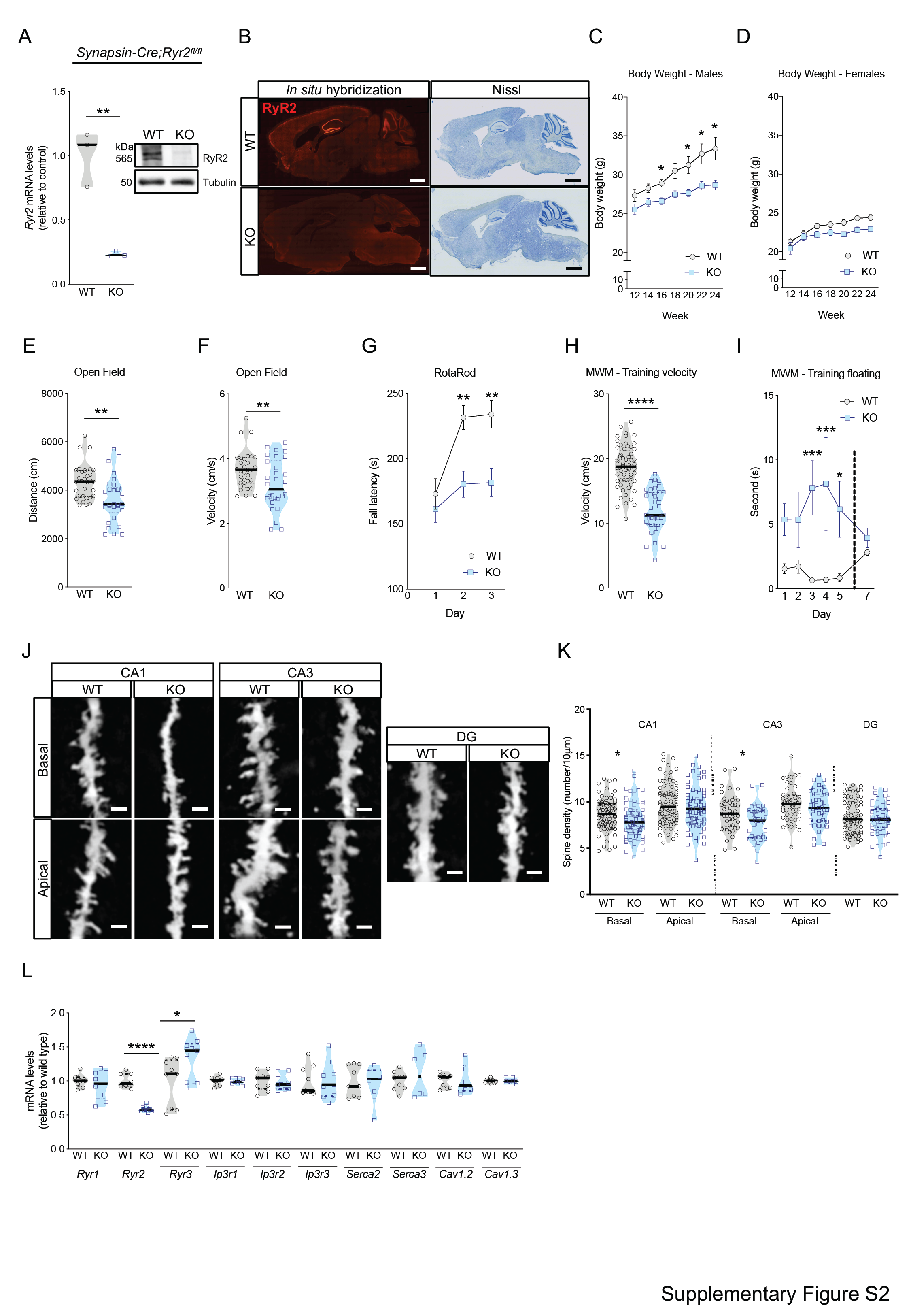

Supplement: Supplementary file 2 — Supplementary Figure S2 [file 41418_2020_584_MOESM2_ESM.png]

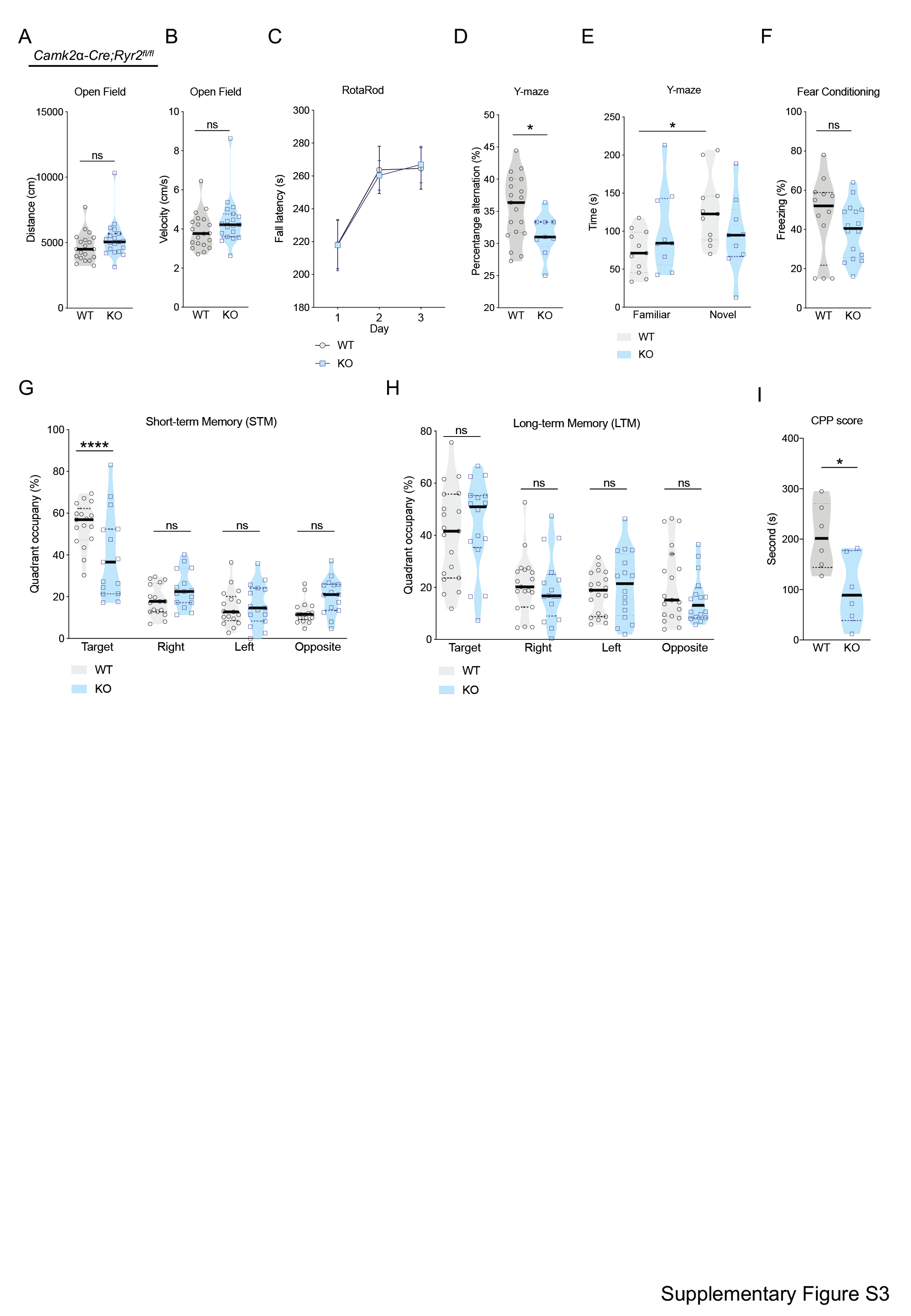

Supplement: Supplementary file 3 — Supplementary Figure S3 [file 41418_2020_584_MOESM3_ESM.png]

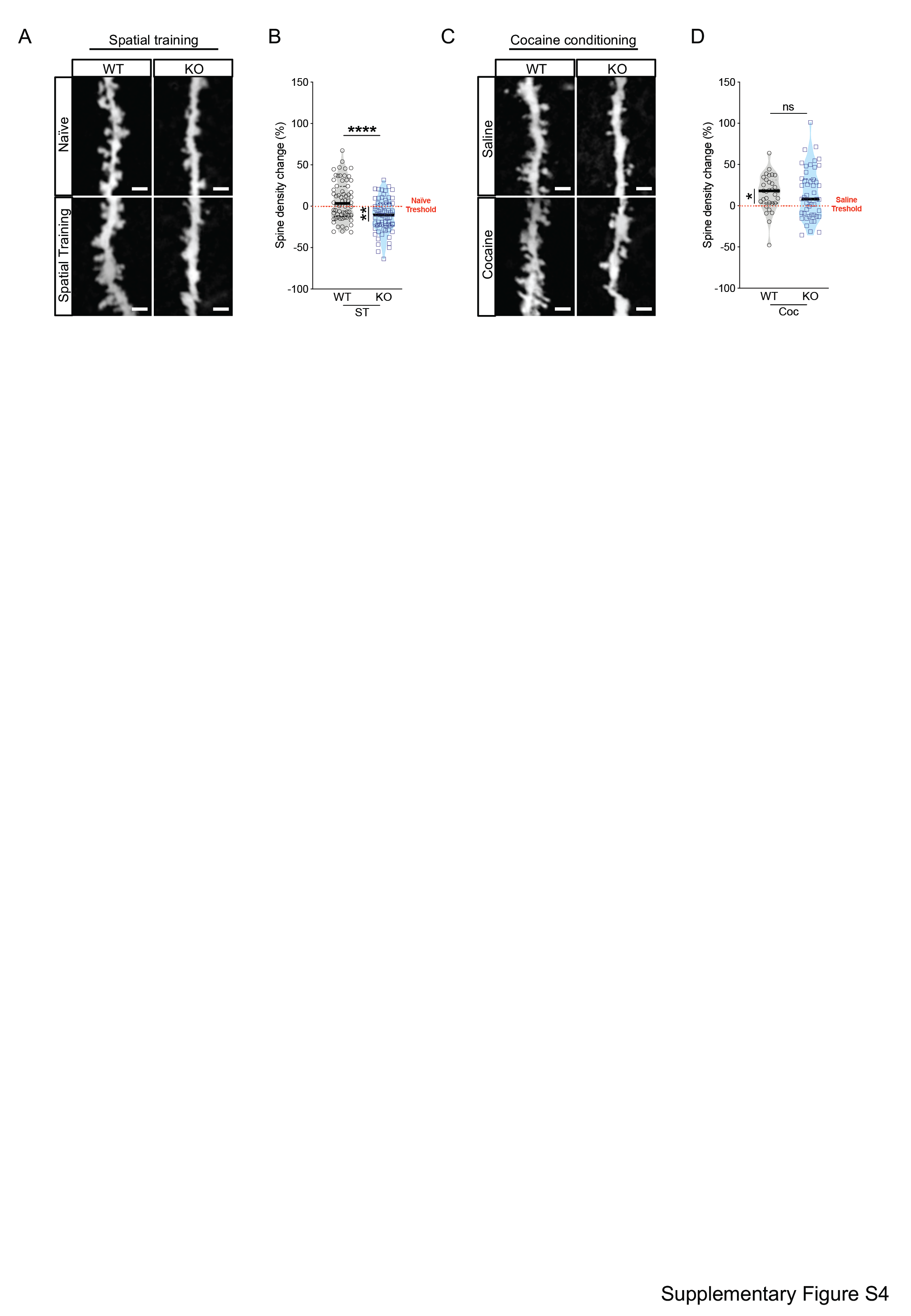

Supplement: Supplementary file 4 — Supplementary Figure S4 [file 41418_2020_584_MOESM4_ESM.png]

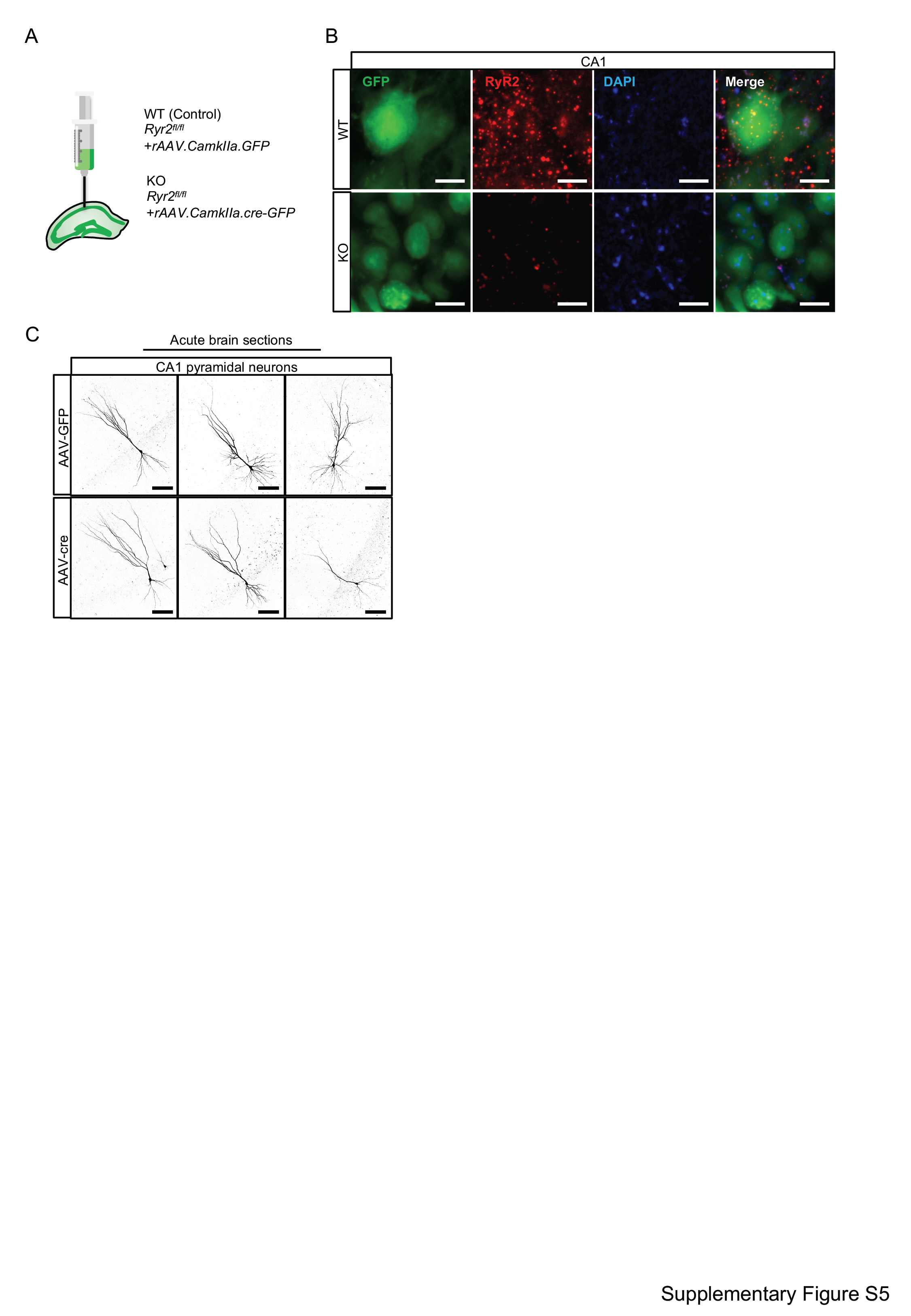

Supplement: Supplementary file 5 — Supplementary Figure S5 [file 41418_2020_584_MOESM5_ESM.png]

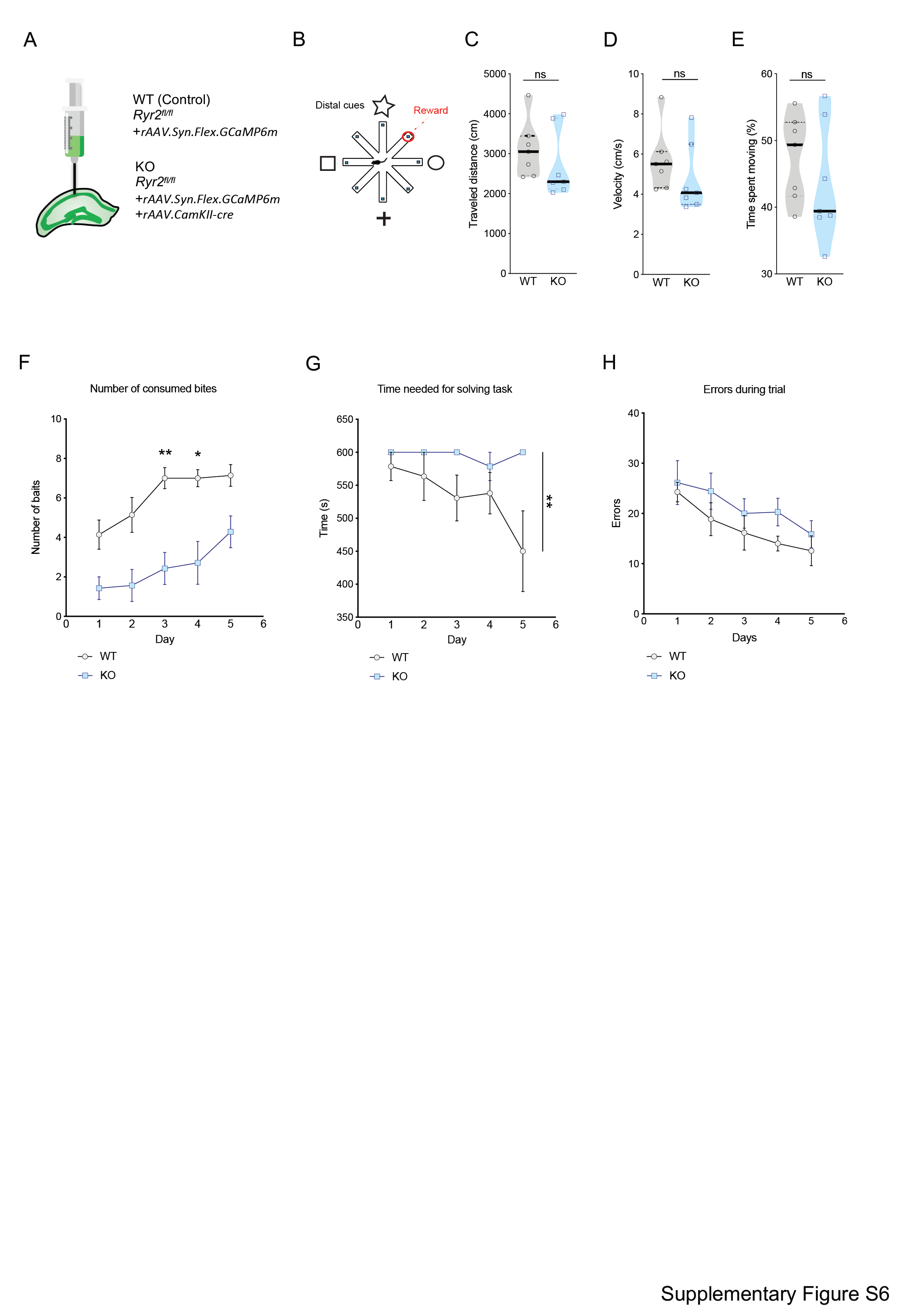

Supplement: Supplementary file 6 — Supplementary Figure S6 [file 41418_2020_584_MOESM6_ESM.png]
