# Supplementary material for: Loss of Ryanodine Receptor 2 impairs neuronal activity-dependent remodeling of dendritic spines and triggers compensatory neuronal hyperexcitability
Source: Cell Death Differ. 2020 Jul 8;27(12):3354–73. doi: 10.1038/s41418-020-0584-2 (PMC7853040; doi:10.1038/s41418-020-0584-2)
Supplement: Supplementary file 7 — Table S1 [file 41418_2020_584_MOESM7_ESM.docx]

**Table S1. Statistical analysis of dendritic spines.**

| DIV14 Primary hippocampal neurons | | | | | | |
| --- | --- | --- | --- | --- | --- | --- |
| **Fig.** | **Model** | **Neurons** | **Test** | **Factor** | **p-value** | **Sum** |
| **1C** | Primary neurons  Ryanodine | DIV14 | Unpaired t test | Genotype | *p*=0.0082 | ****** |
| **1F** | Primary neurons  RyR2 downreg. | DIV14 | Unpaired t test | Genotype | *p*=0.0055 | ****** |
| **2C** | Primary neurons  cLTP | DIV14 | Unpaired t test | Treatment | scr  *p*=0.0005 | ******* |
|  |  |  |  |  | sh  *p*=0.9918 | ns |
|  |  |  |  | Genotype | *p*=0.0009 | ******* |
| *Camk2α- Cre^wt/wt^;Ryr2^fl/fl^* (WT) vs *Camk2α- Cre^tg/wt^;Ryr2^fl/fl^* (KO) mice | | | | | | |
| **Fig.** | **Model** | **Neurons** | **Test** | **Factor** | **p-value** | **Sum** |
| **1I** | *Camk2α- Cre;Ryr2^fl/fl^* | CA1 apical | Unpaired t test | Genotype | *p*=0.057 | ns |
| **1I** | *Camk2α- Cre;Ryr2^fl/fl^* | CA1 basal | Unpaired t test | Genotype | *p*=0.0063 | ** |
| **2G** | *Camk2α- Cre;Ryr2^fl/fl^*  Spatial Training | CA1 apical | Unpaired t test | Treatment | WT  *p*<0.0001 | **** |
|  |  |  |  |  | KO  *p*=0.3911 | ns |
|  |  |  |  | Genotype | *p*<0.0001 | **** |
| **S4B** | *Camk2α- Cre;Ryr2^fl/fl^*  Spatial Training | CA1 basal | Unpaired t test | Treatment | WT  *p*=0.1700 | ns |
|  |  |  |  |  | KO  *p*=0.0092 | ** |
|  |  |  |  | Genotype | *p*<0.0001 | **** |
| **2K** | *Camk2α- Cre;Ryr2^fl/fl^*  Cocaine | CA1 apical | Unpaired t test | Treatment | WT  *p*=0.0061 | ** |
|  |  |  |  |  | KO  *p*=0.1620 | ns |
|  |  |  |  | Genotype | *p*=0.0431 | * |
| **S4D** | *Camk2α- Cre;Ryr2^fl/fl^*  Cocaine | CA1 basal | Unpaired t test | Treatment | WT  *p*=0.0132 | * |
|  |  |  |  |  | KO  *p*=0.0735 | ns |
|  |  |  |  | Genotype | *p*=0.0791 | ns |
| *Synapsin- Cre^wt/wt^;Ryr2^fl/fl^* (WT) vs *Synapsin- Cre^tg/wt^;Ryr2^fl/fl^* (KO) mice | | | | | | |
| **Fig.** | **Model** | **Neurons** | **Test** | **Factor** | **p-value** | **Sum** |
| **S2K** | *Synapsin- Cre;Ryr2^fl/fl^* | CA1 apical | Unpaired t test | Genotype | *p*=0.209 | ns |
| **S2K** | *Synapsin- Cre;Ryr2^fl/fl^* | CA1 basal | Unpaired t test | Genotype | *p*=0.038 | ***** |
| **S2K** | *Synapsin- Cre;Ryr2^fl/fl^* | CA3 apical | Unpaired t test | Genotype | *p*=0.309 | ns |
| **S2K** | *Synapsin- Cre;Ryr2^fl/fl^* | CA3 basal | Unpaired t test | Genotype | *p*=0.014 | ***** |
| **S2K** | *Synapsin- Cre;Ryr2^fl/fl^* | DG | Unpaired t test | Genotype | *p*=0.37 | ns |
