# Supplementary material for: Loss of Ryanodine Receptor 2 impairs neuronal activity-dependent remodeling of dendritic spines and triggers compensatory neuronal hyperexcitability
Source: Cell Death Differ. 2020 Jul 8;27(12):3354–73. doi: 10.1038/s41418-020-0584-2 (PMC7853040; doi:10.1038/s41418-020-0584-2)
Supplement: Supplementary file 8 — Table S2. [file 41418_2020_584_MOESM8_ESM.docx]

**Table S2. Statistical analysis of behavioral test in control vs RyR2 KO mice.**

| *Camk2α- Cre^wt/wt^;Ryr2^fl/fl^* (WT) vs *Camk2α- Cre^tg/wt^;Ryr2^fl/fl^* (KO) mice | | | | | | | | | | |
| --- | --- | --- | --- | --- | --- | --- | --- | --- | --- | --- |
| **Fig.** | **Test** | **Statistic** | **Factor** | **F(DFn,Dfd)** | **p-value** | **Sum** | **Bonferroni** | **p-value** | **Sum** |  |
| 2D | MWM  Escape Latencies | Two-way RM  ANOVA | Interaction  Day  Genotype | F(4,116)=1.003  F(4,116)=34  F(1,29)=9.938 | *p*=0.409  *p*<0.0001  *p*=0.0037 | ns  ****  ** | WT vs KO  Day1  Day2  Day3  Day4  Day5 | *p*>0.9999  *p*=0.0936  *p*=0.0121  *p*=0.1196  *p*>0.9999 | ns  ns  *  ns  ns |  |
| 2H | CCP Cocaine paired chamber | Two-way  ANOVA | Compart. for Genotype  Compart.  Genotype | F(1,11)=5.706  F(1,11)=51.18  F(1,11)=0.23 | *p*=0.035  *p*<0.0001  *p*=0.63 | *  ****  ns | Sal vs Coc  WT  KO | *p*<0.0001  *p*=0.0156 | ****  * |  |
| S3A | Open Field Distance | Unpaired t test | Genotype |  | *p*=0.157 | ns | - | - | - |  |
| S3B | Open Field Velocity | Unpaired t test | Genotype |  | *p*=0.159 | ns |  |  |  |  |
| S3C | RotaRod  Fall latency | Two-way RM  ANOVA | Interaction  Day  Genotype | F(2,58)=0.061  F(2,58)=21.31  F(1,29)=0.0009 | *p*=0.94  *p*<0.0001  *p*=0.97 | ns  ****  ns | - | - | - |  |
| S3D | Y-Maze  Spontaneous alternation | Unpaired t test | Genotype |  | *p*=0.03 | * |  |  |  |  |
| S3E | Y-Maze  Spatial memory | Two-way  ANOVA | Interaction  Arm  Genotype | F(1,36)=3.309  F(1,36)= 2.26  F(1,36)= 0.0162 | *p*=0.0856  *p*=0.1501  *p*=0.9001 | ns  ns  ns | Fam / Novel  WT  KO | *p*=0.046  *p*>0.99 | *  ns |  |
| S3F | Fear conditioning  Retrieval | Unpaired t test | Genotype |  | *p*=0.3022 | ns | - | *-* | - |  |
| S3G | MWM  Quadrant occupancy (90min) | Two-way  RM  ANOVA | Interaction  Quandrant  Genotype | F(3,90)=5.293  F(3,90)=46.28  F(1,90)=1.136 | *p*=0.0021  *p*<0.0001  *p*=0.295 | **  ****  ns | WT vs KO  Target  Right  Left  Opposite | *p*=0.0008  *p*=0.6355  *p*>0.9999  *p*=0.1689 | ***  ns  ns  ns |  |
| S3H | MWM  Quadrant occupancy (24h) | Two-way  RM  ANOVA | Interaction  Quandrant  Genotype | F(3,99)=0.214  F(3,99)=18.5  F(1,33)=2.112 | *p*=0.602  *p*<0.0001  *p*=0.1556 | ns  ****  ns | WT vs KO  Target  Right  Left  Opposite | *p*>0.9999  *p*>0.9999  *p*>0.9999  *p*>0.9999 | ns  ns  ns  ns |  |
| S3I | CCP score  Cocaine treatment | Unpaired t test | Genotype |  | *p*=0.0204 | * | - | *-* | - |  |

| *Synapsin- Cre^wt/wt^;Ryr2^fl/fl^* (WT) vs *Synapsin- Cre^tg/wt^;Ryr2^fl/fl^* (KO) mice | | | | | | | | | | |
| --- | --- | --- | --- | --- | --- | --- | --- | --- | --- | --- |
| **Fig.** | **Test** | **Statistic** | **Factor** | **F(DFn,Dfd)** | **p-value** | **Sum** | **Bonferroni** | **p-value** | **Sum** |  |
| S2C | Body weight  males | Two-way  ANOVA | Interaction  Age  Genotype | F(6,116)=3.309  F(6,116)= 2.26  F(1,116)= 0.016 | *p*=0.2775  *p*<0.0001  *p*<0.0001 | ns  ****  **** | WT vs KO  Week 12  Week 14  Week 16  Week 18  Week 20  Week 22  Week 24 | *p*=0.903  *p*=0.67  *p*=0.301  *p*=0.2257  *p*=0.064  *p*=0.0140  *p*=0.0019 | ns  ns  ns  ns  ns  *****  ****** |  |
| S2D | Body weight  females | Two-way  ANOVA | Interaction  Age  Genotype | F(6,208)=0.457  F(6,208)= 9.215  F(1,208)= 23.04 | *p*=0.8393  *p*<0.0001  *p*<0.0001 | ns  ****  **** | WT vs KO  Week 12  Week 14  Week 16  Week 18  Week 20  Week 22  Week 24 | *p*=0.989  *p*>0.999  *p*=0.762  *p*=0.94  *p*=0.51  *p*=0.51  *p*=0.58 | ns  ns  ns  ns  ns  ns  ns |  |
| S2E | Open Field Distance moved | Unpaired t test | Genotype |  | *p*=0.009 | ******* | **-** | **-** | **-** |  |
| S2F | Open Field Velocity | Unpaired t test | Genotype |  | *p*=0.0091 | ****** | **-** | **-** | **-** |  |
| S2G | RotaRod  Fall latency | Two-way RM  ANOVA | Interaction  Day  Genotype | F(2,120)=6.597  F(2,120)=26.03  F(1,60)=9.041 | *p*=0.002  *p*<0.0001  *p*=0.0039 | ******  ********  ****** | WT vs KO  Day 1  Day2  Day3 | 0.8156  0.0019  0.0015 | ns  **  ** |  |
| S2H | MWM  Training velocity | Unpaired t test | Genotype |  | *p*<0.0001 | ******** | **-** | **-** | **-** |  |
| S2I | MWM  Training floating | Two-way RM  ANOVA | Interaction  Day  Genotype | F(5,85)=3.99  F(5,85)=0.55  F(1,17)=13.44 | *p*=0.002  *p*=0.73  *p*=0.0019 | ******  ns  ****** | **-** | **-** | **-** |  |
| *Ryr2^fl/fl^; +AAV.Syn.Flex.GCaMP6m* (WT) vs *Ryr2^fl/fl^; +AAV.Syn.Flex.GCaMP6m*; +rAAV.CamkII-cre (KO) mice | | | | | | | | | |  |
| **Fig.** | **Test** | **Statistic** | **Factor** | **F(DFn,Dfd)** | **p-value** | **Sum** | **Bonferroni** | **p-value** | **Sum** |  |
| S6C | Radial Arm Maze  Distance moved | Unpaired t test | Genotype | - | *p*=0.36 | ns | - | - | - |  |
| S6D | Radial Arm Maze  Velocity | Unpaired t test | Genotype | - | *p*=0.311 | ns | - | - | - |  |
| S6E | Radial Arm Maze  Time spent moving | Unpaired t test | Genotype | - | *p*=0.347 | ns | - | - | - |  |
| S6F | Radial Arm Maze  Number of consumed bites | Two-way RM  ANOVA | Day x Gen.  Day  Genotype | F(4,48)=1.17  F(2.9,35.1)=  9.68  F(1,12)=5.547 | *p*=0.333  *p*<0.0001  *p*=0.0008 | ns  ****  ******* | WT vs KO  Day1  Day2  Day3  Day4  Day5 | *P*=0.069  *P*=0.058  *p*=0.0038  *p*=0.0325  *p*=0.0725 | ns  ns  **  *  ns |  |
| S6G | Radial Arm Maze  Time solving Task | Two-way RM  ANOVA | Day x Gen.  Day  Genotype | F(4,48)=1.985  F(2.1,24.9)=  1.89  F(1,12)=7.032 | *p*=0.119  *p*=0.1707  *p*=0.0211 | ns  ns  ***** | WT vs KO  Day1  Day2  Day3  Day4  Day5 | *p*>0.9999  *p*>0.9999  *p*=0.46  *p*>0.9999  *p*=0.24 | ns  ns  ns  ns  ns |  |
| S6H | Radial Arm Maze  Errors | Two-way RM  ANOVA | Day x Gen.  Day  Genotype | F(4,48)=3.99  F(2.8, 34.2)= 7.054  F(1,12)=1.669 | *p*=0.871  *p*=0.001  *p*=0.2207 | ns  **  ns | WT vs KO  Day1  Day2  Day3  Day4  Day5 | *p*>0.9999  *p*>0.9999  *p*=0.46  *p*>0.9999  *p*=0.24 | ns  ns  ns  ns  ns |  |
