# Supplementary material for: Loss of Ryanodine Receptor 2 impairs neuronal activity-dependent remodeling of dendritic spines and triggers compensatory neuronal hyperexcitability
Source: Cell Death Differ. 2020 Jul 8;27(12):3354–73. doi: 10.1038/s41418-020-0584-2 (PMC7853040; doi:10.1038/s41418-020-0584-2)
Supplement: Supplementary file 9 — Supplementary Figure Legends [file 41418_2020_584_MOESM9_ESM.docx]

**Supplementary Figure S1. Validation of RyR2 knockout transgenic mice. (**A) Schematic representation of the gene editing strategy for the deletion of *Ryr2*. Two loxP sites were inserted flanking exon 8 in the *Ryr2* locus allowed the Cre-recombination resulted in the insertion of a STOP codon. (B) Ubiquitous deletion of *Ryr2* resulted in embryonic lethality of *CMV-Cre^tg/wt^;Ryr2^fl/fl^* mice at E12.5 (WT: n=3, KO n=3). Scale bar: 1 mm. **(**C) Southern blot of *Ryr2* gene in control (WT) and whole-brain *Nestin-Cre^tg/wt^;Ryr2^fl/fl^* (KO) mice (WT: n=3, KO n=3).

**Supplementary Figure S2. *Synapsin-Cre^tg/wt^;Ryr2^fl/fl^* mice show decreased spine density in CA1 and CA3 of the hippocampus.** (A) qRT-PCR and western blot of RyR2 using brain homogenates from controls (WT) and *Synapsin-Cre^tg/wt^;Ryr2^fl/fl^* (KO) mice (WT: n=3, KO: n=3; unpaired t-test, *Ryr2* *p*=0.0036). (B) *In situ* hybridization for *Ryr2* and Nissl staining in sagittal brain sections from control (WT) and *Synapsin-Cre^tg/wt^; Ryr2^fl/fl^* (KO) mice*.* Scale bar 1 mm. (C) Body weight measurement in control (WT) and *Synapsin-Cre^tg/wt^;Ryr2^fl/fl^* (KO) males (WT: n=7, KO: n=9). (D) Body weight measurement in control (WT) and *Synapsin-Cre^tg/wt^; Ryr2^fl/fl^* (KO) females (WT: n=11, KO: n=19). (E) Travelled distance measured in the open field of control (WT) and *Synapsin-Cre^tg/wt^;* *Ryr2^fl/fl^* (KO) mice (WT: n=20, KO: n=19). (F) Mean velocity measured in the open field of control (WT) and *Synapsin-Cre^tg/wt^;* *Ryr2^fl/fl^* (KO) mice (WT: n=20, KO: n=19). **(**G) Fall latencies was measured in the rotarod test comparing control (WT) and *Synapsin-Cre^tg/wt^;Ryr2^fl/fl^* (KO) mice (WT: n=20, KO: n=19). (H) Swim speed and (I) floating time during the training in the Morris Water Maze of control (WT) and *Synapsin-Cre^tg/wt^;Ryr2^fl/fl^* (KO) mice (WT: n=12, KO: n=7). (J-K) Representative pictures and quantifications of spine density in neurons from CA1 (WT: n=82 cells/ mice n=7, KO: n=71 cells/ mice n=6), CA3 (WT: n=44 cells/ mice n=7, KO: n=38 cells/ mice n=6) and DG (WT: n=75 cells/ mice n=7, KO: n=56 cells/ mice n=6) comparing control (WT) and *Synapsin-Cre^tg/wt^;Ryr2^fl/fl^* (KO) mice. Scale bar: 2 μm. (L) qRT-PCR analysis of channels involved in Ca^2+^ homeostasis from dissected hippocampal samples in control (WT) and *Synapsin-Cre^tg/wt^;Ryr2^fl/fl^* (KO) mice (WT: n=9, KO: n=9; unpaired t-test, *Ryr2* *p*=0.0033, *Ryr3 p*=0.0456). Data are reported as median [25^th^ and 75^th^ percentile] or mean ± SEM. qRT-PCR values are 2^−ΔΔCT^; Unpaired Student’s t test or RM Two-way ANOVA with Bonferroni post hoc comparison, *****p*<0.0001, ****p*<0.001, ***p*<0.01, **p*<0.05.

**Supplementary Figure S3. *Camk2α-Cre^tg/wt^; Ryr2^fl/fl^* mice show impaired working, short-term and context-associated memories.** (A) Distance run in open field test of control (WT) and *Camk2α-Cre^tg/wt^;Ryr2^fl/fl^* (KO) mice (WT: n=20, KO: n=19). (B) Mean run velocity in open field test of control (WT) and *Camk2α-Cre^tg/wt^;Ryr2^fl/fl^* (KO) mice (WT: n=20, KO: n=19). (C) Fall latency of control (WT) and *Camk2α-Cre^tg/wt^;Ryr2^fl/fl^* (KO) mice in RotaRod test (WT: n=15, KO: n=16). (D) Spontaneous alternations of control (WT) and *Camk2α-Cre^tg/wt^;Ryr2^fl/fl^* (KO) mice in the Y-maze test (WT: n=19, KO: n=9). (E) Time spent in the familiar and novel arm of control (WT) and *Camk2α-Cre^tg/wt^;Ryr2^fl/fl^* (KO) mice in the Y-maze test (WT: n=11, KO: n=9). (F) Contextual fear conditioning freezing time of control (WT) and *Camk2α-Cre^tg/wt^;Ryr2^fl/fl^* (KO) mice (WT: n=12, KO: n=16). (G-H) Control (WT) and *Camk2α-Cre^tg/wt^;Ryr2^fl/fl^* (KO) mice were subjected to Morris Water Maze. (G) Short-term memory was tested in control (WT) and *Camk2α-Cre^tg/wt^;Ryr2^fl/fl^* (KO) mice measuring the target quadrant occupancy at day 3 (WT: n=13, KO: n=18). (H) Long-term memory was tested in control (WT) and *Camk2α-Cre^tg/wt^;Ryr2^fl/fl^* (KO) mice measuring the target quadrant occupancy 24 h after the last training at day 6 (WT: n=12, KO: n=16). (I) Contextual place preference score of cocaine-treated control (WT) versus *Camk2α-Cre^tg/wt^;Ryr2^fl/fll^* (KO) mice 24 h after the last cocaine conditioning (WT cocaine: n=8, KO cocaine: n=8). Data are reported as median [25^th^ and 75^th^ percentile] or mean ± SEM. Unpaired Student’s t test or RM Two-way ANOVA with Bonferroni post hoc comparison, *****p*<0.0001, **p*<0.05.

**Supplementary Figure S4. Activity-dependent structural plasticity of dendritic spines in basal dendrites of CA1 cells.** (A-B) Representative pictures and quantification of spine density change in basal dendrites of CA1 neurons in Morris Water Maze spatially trained control (WT) and *Camk2α-Cre^tg/wt^;Ryr2^fl/fl^* (KO) mice compared to naïve mice (WT naïve: n=48 cells/ 5 mice, WT trained: n=66 cells/ 7 mice, KO naïve: n=50 cells/ 5 mice, KO trained: n=67 cells/ 7 mice). (C-D) Representative pictures and quantification of spine density change 24 h after the post-conditioning test in basal (WT naïve: n=24 cells/ 3 mice , WT cocaine: n=31 cells/ 4 mice, KO naïve: n=32 cells/ 4 mice, KO cocaine: n=48 cells/ 6 mice) dendrites of CA1 neurons, comparing cocaine-treated control (WT) and *Camk2α-Cre^tg/wt^;Ryr2^fl/fl^* (KO) mice to saline-injected mice. Scale bar: 2 μm. Data are reported as median [25^th^ and 75^th^ percentile]. Unpaired Student’s t test. *****p*<0.0001.

**Supplementary Figure S5. Adeno associated viral (AAV) deletion of RyR2 in CA1 pyramidal neurons.** (A) *Ryr2^fl/fl^* mice were stereotaxically injected with *AAV.Camk2a.GFP*, as a control (WT), or with *rAAV.Camk2a.GFP-cre* (KO) to delete RyR2 in the CA1 of the hippocampus. (B) *In-situ* hybridization of *Ryr2* mRNA in control (WT) and RyR2 knockout (KO) CA1 cells. Scale bar: 10 μm. (C) Representative reconstructions of recorded control (WT) and RyR2 knockout (KO) CA1 pyramidal neurons. Scale bar: 100 μm.

**Supplementary Figure S6. Radial arm maze behavioural test.** (A) Injection of combination of AAVs into hippocampus of right hemisphere. Radial arm maze of *Ryr2^fl/fl^* mice stereotactically injected with r*AAV1.Syn.Flex.GCaMP6m* as a control (WT) or with *AAV9.CamKII.cre*. and r*AAV1.Syn.Flex.GCaMP6m* to delete RyR2 (KO). (B) Top-view on arena. (C) Travelled distance of control (WT) and RyR2 knockout (KO) mice on day 1 of testing (WT: n=7, KO: n=7). (D) Average velocity of control (WT) and RyR2 knockout (KO) mice on day 1 of testing (WT: n=7, KO: n=7). (E) Average relative time spent moving of control (WT) and RyR2 knockout (KO) mice on day 1 of testing (WT: n=7, KO: n=7). (F) Average number of consumed baits of control (WT) and RyR2 knockout (KO) mice at the end of each test day. (WT: n=7, TG n=7). (G) Average time required for clearance of all baits in control (WT) and RyR2 knockout (KO) mice (WT: n=7, KO n=7). (H) Average number of errors of control (WT) and RyR2 knockout (KO) mice (arm-entries without consuming bait) during each trial day (WT: n=7, KO n=7). Data are reported as median [25^th^ and 75^th^ percentile] or mean ± SEM. Data are reported as median [25^th^ and 75^th^ percentile] or mean ± SEM. Unpaired Student’s t test or RM Two-way ANOVA with Bonferroni post hoc comparison. (****p*<0.001, ***p*<0.01, **p*<0.05).
